# Sleep Quality Changes during Overwintering at the German Antarctic Stations Neumayer II and III: The Gender Factor

**DOI:** 10.1371/journal.pone.0150099

**Published:** 2016-02-26

**Authors:** Mathias Steinach, Eberhard Kohlberg, Martina Anna Maggioni, Stefan Mendt, Oliver Opatz, Alexander Stahn, Hanns-Christian Gunga

**Affiliations:** 1 Center for Space Medicine and Extreme Environments Berlin, Institute for Physiology, Charité Universitätsmedizin Berlin, Berlin, Germany; 2 Alfred Wegener Institute for Polar and Marine Research, Bremerhaven, Germany; 3 Department of Biomedical Sciences for Health, Università degli Studi di Milano, Milan, Italy; University of Würzburg, GERMANY

## Abstract

**Purpose:**

Antarctic residence holds many challenges to human physiology, like increased psycho-social tension and altered circadian rhythm, known to influence sleep. We assessed changes in sleep patterns during 13 months of overwintering at the German Stations Neumayer II and III from 2008 to 2014, with focus on gender, as many previous investigations were inconclusive regarding gender-based differences or had only included men.

**Materials & Methods:**

Time in bed, sleep time, sleep efficiency, number of arousals, sleep latency, sleep onset, sleep offset, and physical activity level were determined twice per month during seven overwintering campaigns of n = 54 participants (37 male, 17 female) using actimetry. Data were analyzed using polynomial regression and analysis of covariance for change over time with the covariates gender, inhabited station, overwintering season and influence of physical activity and local sunshine radiation.

**Results:**

We found overall longer times in bed (p = 0.004) and sleep time (p = 0.014) for women. The covariate gender had a significant influence on time in bed (p<0.001), sleep time (p<0.001), number of arousals (p = 0.04), sleep latency (p = 0.04), and sleep onset (p<0.001). Women separately (p = 0.02), but not men (p = 0.165), showed a linear increase in number of arousals. Physical activity decreased over overwintering time for men (p = 0.003), but not for women (p = 0.174). The decline in local sunshine radiation led to a 48 minutes longer time in bed (p<0.001), 3.8% lower sleep efficiency (p<0.001), a delay of 32 minutes in sleep onset (p<0.001), a delay of 54 minutes in sleep offset (p<0.001), and 11% less daily energy expenditure (p<0.001), for all participants in reaction to the Antarctic winter’s darkness-phase.

**Conclusions:**

Overwinterings at the Stations Neumayer II and III are associated with significant changes in sleep patterns, with dependences from overwintering time and local sunshine radiation. Gender appears to be an influence, as women showed a declining sleep quality, despite that their physical activity remained unchanged, suggesting other causes such as a higher susceptibility to psycho-social stress and changes in environmental circadian rhythm during long-term isolation in Antarctica.

## Introduction

Life and work of humans in high latitudes, i.e. latitudes close to polar regions such as the Arctic or Antarctic, are often associated with adverse conditions such as very cold climate, changed circadian cycle and altered exposure to ultra-violet (UV)-light [1–3]. In addition, human stays in Antarctic research stations may be associated with psycho-social isolation, sensory deprivation and exhaustion [4,5]–a combination of adverse factors, which subsequently may lead to challenges to hormonal, metabolic and immune functions [6,7]. Polar regions as the Antarctic receive less intensive solar radiation because the sun’s light hits the Earth at an oblique angle. In addition, Antarctica’s climate is dominated by seasonal changes. Depending on latitude, months of complete darkness during the Antarctic winter alternate with months of 24-hours bright daylight during the Antarctic summer [8], leading to altered lighting conditions known to negatively affect the circadian cycle of human residents in Antarctica [9]. The nature of stays in polar regions, such as during long-term isolation in Antarctic research stations, has led to consider such overwinterings to be viable analogues to long-term spaceflights [10,11].

The environmental and psycho-social influences of long-term stays in the Antarctic seem to have particular consequences on sleep homeostasis for humans residing there. Several investigations have reported changes in sleep pattern of Antarctic overwinterers [3,12,13], with reduction in sleep quality, such as decrease in delta sleep phases and rapid eye movement phases as well as increased sleep latency [14–16]. Reduction in sleep quality has been linked to the influence of environmental factors as well as psycho-social reactions to the isolation [17,18]. Decreases in sleep time and sleep efficiency, and increased sleep latency, have also been found to prevail in a modern Antarctic station [19], with reports that application of blue-enriched light in such a setting may prevent circadian delay during the Antarctic winter [20]. There are, however, also studies that did not observe changes in sleep pattern during long-term stays in Antarctica, suggesting that modern research stations in the Antarctic may be less prone to changes in sleep of their inhabitants [21,22]. The majority of previous studies had reported to have only included men as their participants [12–17,19,22], thus indicating the need to gather more information regarding gender-based differences in this field, as has previously been summarized [9].

Germany currently runs the year-long inhabited research station Neumayer III, which is in operation since February 2009; it replaced the previous station Neumayer II, which operated from 1992 to 2009 and which had to be abandoned, since its structural integrity, while being located underground within the ice, could no longer be maintained [23]. Neumayer Stations II and III served as the sites of the presented study. Their remote location and the very small crew size of less than 10 persons per overwintering season were considered to lead to a relatively high degree of isolation, when compared to other Antarctic facilities such as McMurdo Station, where the winter population can reach around 250 inhabitants [24].

The aim of this study was to assess changes in sleep parameters in overwinterers of the isolated German Research Stations Neumayer II and III for 13 months during a total of seven campaigns from 2008 to 2014. In addition, only few previous studies regarding sleep changes during long-term isolation in Antarctica had included women, some of which had either yielded inconclusive results [3] or indicated that women have more sleep problems during Antarctic isolation than men with a considerable number of study participants, but were based on debriefing interviews only [25]. We consider gender-specific investigations to become more important in the future as gender-barriers fall and more women participate in ventures to extreme environments such as polar expeditions or space missions [26], fields that used to be dominated by men in the past. We therefore intended to investigate the influence of gender since our long-term study included a considerable number of women. Our null hypothesis was that sleep parameters would not change during the Antarctic winter and that gender, inhabited station (located below vs. above ground), overwintering season, physical activity, and local sunshine radiation would not have a significant influence on the measured sleep parameters.

## Materials and Methods

### General circumstances

German Antarctic research stations Neumayer Station II and Neumayer Station III, operated by the “Alfred Wegener Institute for Polar and Marine Research” (Bremerhaven, Germany), were the location of the investigation. Station II was and station III is located in Atka-bay 70° 40’ S, 8° 16’ W on the Ekström-shelf ice [23]. The inhabitants of the Neumayer Stations were subject to considerable isolation due to the station’s remote location, nature of inaccessibility, and small crew size per overwintering [23]. Adverse weather conditions during the darkness-phase made it nearly impossible to reach the station by airplane or ships, which led to complete isolation of the inhabitants during the months of the Antarctic winter; merely an Internet connection and different satellite communication systems allowed contact with the outside world. Emergency rescues would have been practically impossible [23].

Stations Neumayer II and III serve to gather data in aerial-chemical, geophysical and meteorological investigations, and, since the beginning of 2000, also for medical and physiological studies. Station Neumayer II was located underground and consisted of two tube constructions (90-m in length and 8 m in diameter) connected to each other. Air-conditioned standard 20-foot containers served as laboratories, workshops, sickbay, etc. In a third tube chill camps, garbage storage as well as the vehicle garage were accommodated [23]. Since February 2009, the new Neumayer Station III is in operation. This is the first German Antarctic station that combines human stay and research on a platform above the ice surface, with a garage built within the ice. This station has the quality to lift itself up according to snow accumulation by the use of hydraulic technics attached to the supporting feet [23]. Neumayer III offers modern air-conditioned laboratories and accommodation areas as well as a sauna, a dining room, a conference room, medical treatment rooms, and storage and technical rooms.

The crews of the Neumayer Stations II and III consisted of employees of different fields and professions (meteorologists, chemists, geophysicists, electricians, engineers, computer technicians, a cook, and a medical doctor, who also acted as commander of the crew). The members of each overwintering crew resided at the Antarctic station for 13 months (from December prior, to January past the respective overwintering year). The recruitment of each crew was carried out by the “Alfred Wegener Institute for Polar and Marine Research” [23]. Aside from their respective fields of occupation, all crewmembers had to tend to duties associated with station maintenance. Due to the risks involved (injuries due to fall, frostbite and hypothermia), the outside-activity during the Antarctic winter was reduced to a minimum; however, each crewmember was equipped with cold-protection clothing and emergency equipment [23]. Supplies were transported to the station once a year by the research vessel "Polarstern". The supply of food to the Neumayer Stations II and III was conducted in accordance with months of inaccessibility. Fresh fruit and vegetables were only available for a short period during summer season from November until February. A rationing was not performed and no restrictions of caloric intake were applied [23].

For the purpose of this study, seven overwintering seasons from 2008 to 2014 were investigated (2008 Neumayer Station II and 2009 to 2014 Neumayer Station III). Per overwintering season, nine adult crewmembers of Caucasian descent lived and worked at the station. The recruitment process of the “Alfred Wegener Institute” included a medical and psychological screening and ensured that crew members were of good physical and mental health and were not taking any medication (except for oral contraceptives by the female participants). During their recruitment, they were invited to participate in this prospective study. One person each in 2008, 2010, 2011, and 2014 decided not to partake, as did two individuals in 2009, and three in 2012. Thus from a total of 63 participants of the seven overwintering campaigns, a total of 54 (37 male and 17 female) took part in the study. The percentage of women differed between overwintering seasons and ranged from 22% in 2010 to 44% in 2013. After the subjects were explained the risks and details of the study, they were given due time to express their desire to participate, they all gave their written informed consent. The study was approved by the local Ethics Committee of Charité Universitätsmedizin Berlin. All procedures were conducted in accordance of the Declaration of Helsinki regarding human subjects.

### Environmental factors

**Fig 1:**
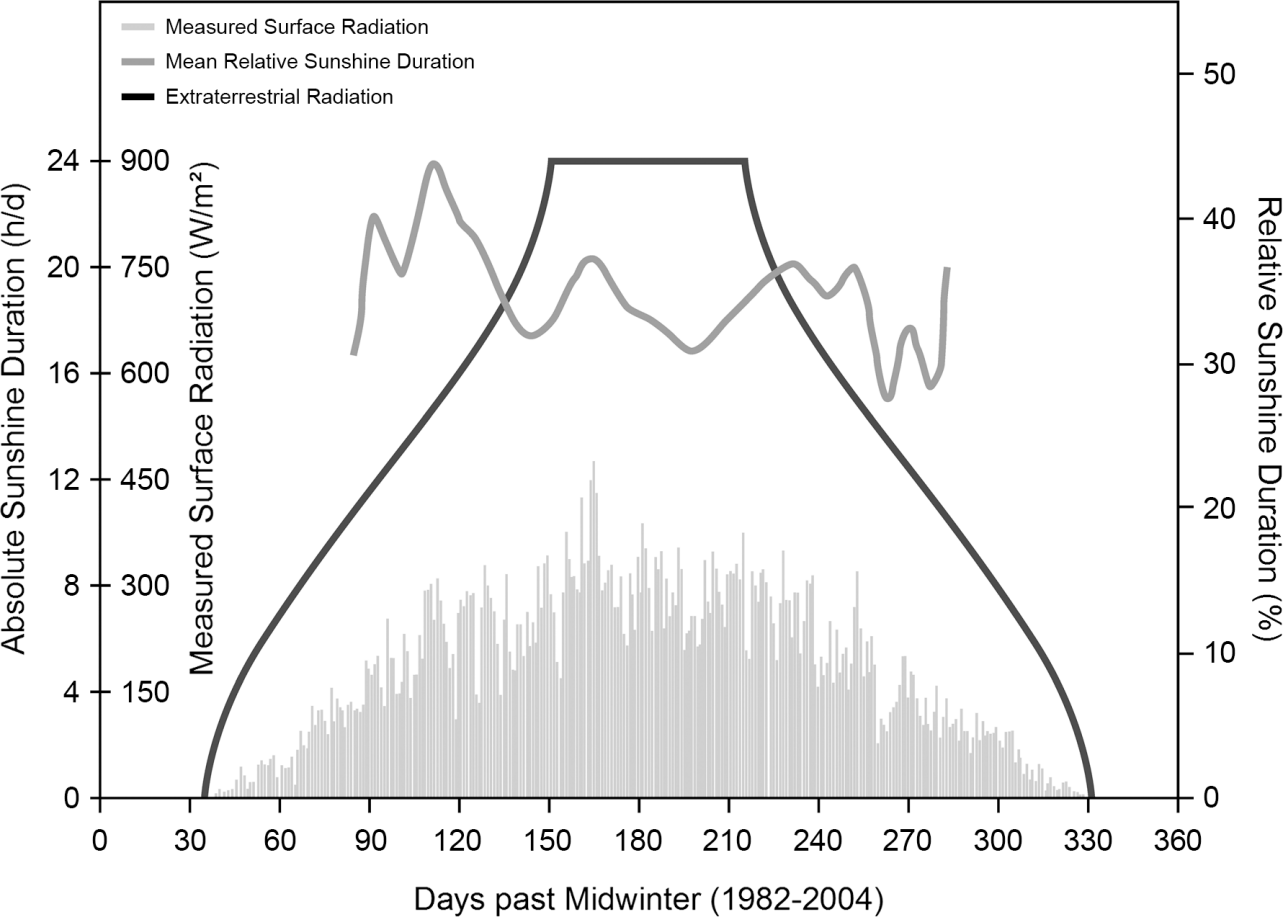
Duration of sunshine at the Neumayer Stations in Antarctica Plots of extraterrestrial radiation (black), relative sunshine duration (dark grey) and measured surface radiation (light grey) at the location of the Neumayer Stations The x-axis displays the time in days with “0” representing midwinter (June 21^st^).

The location of the research stations at 70° south determined the amount of sunshine that reached the surface [8]. As indicated in Fig 1, for a period of about 60 days around midwinter (21^st^ of June), virtually no sunlight reached locations at that latitude, while for another 20 days before and after that period the sunshine radiation was very low, which led to periods of complete darkness between the end of May and the end of July. Less than 50 W/m^2^ of sunshine radiation were measured between beginning of April until the end of September, and less than 5 W/m^2^ from the mid of May until the beginning of August, respectively. Sunshine duration (with the respective low energies) was less than about 5 h/d from mid-April to end of August and virtually zero from mid-May to the beginning of August. Sunshine from November to January during the Antarctic summer, however, can reach up to 24 h/d with 300 to 450 W/m^2^ [23,27]. This also led to very low temperatures. At 12:00 p.m., the mean ambient temperature at the Neumayer Stations between 2008 to 2011 ranged around –2.7 ± 2.0°C (n = 124) and –25.2 ± 7.2°C (n = 124) in January and July, respectively [23].

Although no continuous measurements were made regarding individual light exposition that could have been included in this analysis, there was one measurement of illuminance conducted inside the lounge of the Neumayer Station III at five different times over three days in February 2011 (February 19^th^ at 07:00 p.m., February 20^th^ at 08:00 a.m. and 03:30 p.m., February 21^st^ at 03:45 p.m. and 07:30 p.m.). The local sunshine radiation values at these occasions were 109, 370, 438, 229, and 52.5 W/m^2^. The illuminance measurements used the lux meter MINILUX (MX-ELECTRONIC, Berlin, Germany), which was placed horizontally at 25 different positions inside the lounge of Neumayer Station III approximately 1 m above the floor and 2 m away from each other, while all artificial illumination was switched off. The determined values were in median 330 lux (165 lux 25^th^ percentile, 670 lux 75^th^ percentile) with a minimum of 30 lux and a maximum of 4400 lux. Linear regression analysis between illuminance (denoted as y in lux) and local sunshine radiation at the respective measurement times (denoted as x in W/m^2^) yielded the following relationship:

$$y=1.193\;*\;x+257.1,\;R=0.263,\;R^{2}=0.07,\;(p=0.003).$$

No measurements regarding the spectral distribution of sunlight reaching the inside of the station nor regarding the quantity and quality of the station’s artificial illumination could be performed.

### Determination of anthropometric data

Anthropometric data of the study participants were gathered using standard equipment (medical scale and height meter, SECA, Germany) in minimal clothes. Baseline values of percentage of fat mass from body weight were determined using bioelectrical impedance analysis (BIA 101, AKERN, Italy) for campaigns 2008–2011, using the appropriate prediction formula [28], and whole body plethysmography (BODPOD, COSMED, USA) for campaigns 2012–2014. Both are established methods for determining body composition [29,30].

### Determination of sleep parameters and physical activity level

Actimeters are mobile devices designed to measure acceleration data of the wearer, and thus allow assessment of physical activity, energy expenditure, and sleep parameters [31,32]. Benefits of the use of actimeters are ease of application, low cost, and relatively high acceptance and compliance on the part of the subjects [33]. Since the 1980s, actimeters are used for assessment of sleep in humans. Studies comparing actimetry and polysomnography (PSG) have shown that an agreement of 91–93% could be achieved [34]. Actimetry allows the distinction between sleep and wake state and offers sufficiently reliable information regarding sleep habits and sleep disorders of examined patients [35]. However, inter-individual differences can be very high and may not allow individual prediction of sleep stages [36]. Further disadvantages are less precision of measurements taken and the dependence on subject compliance to adhere to measurement protocol [37].

The actimeters SENSEWEAR (“PRO3” and “MF” versions) used in this study were manufactured by BODYMEDIA, Pittsburgh, PA, USA. This type of actimeter has been validated to reliably measure sleep parameters such as total sleep time, wakes after sleep onset, and sleep efficiency against PSG measurements [38]. Another study with obstructive sleep apnea patients found the estimation of sleep by the device to be in high agreement with PSG, while the estimation of wake was less accurate [39]. Sleep measurements with adolescent subjects showed the device to measure sleep parameters of groups reliably when compared to PSG, however, being less accurate at an individual level [40,41]. Regarding the estimation of energy expenditure and physical activity, it was shown that the device reliably measures physical activity levels in various settings [42–44]. However, during exercise of high intensities, the device appears to underestimate energy expenditure [45]. The device is worn on the back of the right (PRO3) and left upper arm (MF) respectively and detects physiological data including motion by measurement of the acceleration, heat flow, skin temperature, near-body temperature, and skin conductance, according to the manufacturer [46]. Data were averaged and recorded by the device with a frequency of 1/min. Both types (PRO3 and MF) were used simultaneously in our study and applied always by the responsible crew physician, who had been properly instructed prior to departure to Antarctica. Measurement data were analyzed using the manufacturer’s software (professional version 7.0) [46].

All clocks on the Neumayer Stations, and thus all used equipment to measure sleep parameters (actimeters, computers to configure the devices and to readout the measurement data), were set to follow coordinated universal time (UTC). Changes imposed by daylight saving time were not applied on neither of the two stations.

The following sleep parameters of the participants were evaluated: time in bed (duration of time at rest but not at sleep), sleep time (duration of time at sleep), sleep efficiency (ratio between sleep time and time in bed), number of arousals (number of wake events per night), sleep latency (duration of time in bed until sleep onset), sleep onset (time of the day of sleep onset), and sleep offset (time of the day of sleep offset). Data on time in bed and sleep time were calculated through the proprietary software algorithm. The parameter sleep efficiency was calculated subsequently from the data on time in bed and sleep time. The number of arousals as well as the duration of sleep latency and the times of sleep onset and offset were determined manually. Sleep onset and offset are reported as values of the time of the day in hours and minutes, a.m. or p.m. respectively. For the regression analyses of sleep onset, values are reported as number of minutes with “0” referring to 12:00 a.m. (midnight) of the same day or the previous day, depending on whether participants fell asleep: before or after 12:00 a.m., e.g. a sleep onset of 10:00 p.m. would be equal to 1320 min while a sleep onset of 01:00 a.m. would be equal to 1500 min. Regarding the regression analyses of sleep offset, values are reported as number of minutes with “0” referring to 12:00 a.m. (midnight) of the same day the participants woke up, e.g. a sleep offset of 07:00 a.m. would be equal to 420 min.

In addition, we also report the daily physical activity level obtained during the measurements (averaged per 24 hours), measured in MET with one MET representing a metabolic rate equivalent to an oxygen consumption of 3.5 ml O_2_ / kg body weight / minute.

### Times of measurement

The measurements were scheduled to be taken twice per month. However, amount and frequency of the measurements varied between overwintering seasons due to different operational constraints, so that the measurement results were assigned to their nearest two-week time slot for the purpose of analysis, see Table 1. During the overwintering seasons 2008 to 2011, 24-hour measurements using the SENSEWEAR-devices were conducted by the overwintering-crews. During the overwintering seasons 2012 to 2014, 48-hour measurements were performed by the overwintering-crews and the acquired individual data of all measured parameters were subsequently each averaged to a 24-hour period to be compatible with the 24-hour measurements for further analysis.

**Table 1:** Number of participants and number and times of measurement.

		Times of measurement																									
Campaign	n	Jan		Feb		Mar		Apr		May		Jun		Jul		Aug		Sep		Oct		Nov		Dec		Jan^†^	
2008	8	•	•	•	•		•	•	•	•	•	•	•	•	•	•	•	•	•	•	•	•	•	•		•	
2009	7	•	•	•	•	•	•	•	•	•	•	•	•	•	•	•	•	•	•	•	•	•	•	•			
2010	8							•	•	•	•	•	•	•	•	•	•	•	•	•	•	•	•	•	•		•
2011	8			•		•			•		•		•	•	•		•		•		•		•				
2012	6					•		•		•		•		•		•											
2013	9					•		•		•		•		•		•	•			•		•					
2014	8				•		•	•	•		•		•		•		•	•		•		•					
n	54	15	13	21	23	29	21	37	27	35	34	34	35	35	33	34	42	14	27	36	29	26	25	12	3	3	6

Number of total participating overwinterers per campaign (column two) and number of respective times of measurement per overwintering season and month of measurement on a two-week basis according to operational demands of the crews

^†^ denotes the January at the end of the overwintering.

According to the “Alfred Wegener Institute”, there were no specific wake up times imposed on the overwinterers, merely a core work time between 09:00 a.m. and 06:00 p.m on workdays was laid out. Workdays were considered to be Mondays to Saturdays. Thus, the participants were free to sleep longer only on Sundays, with a brunch prepared and consumed by the entire crew usually around 10:00 a.m. There was no mandatory night- or shift-work as all necessary duties were carried out within the core work time, with the only exception of the meteorologist, who had to tend to his or her measurement devices until midnight and then again at 06:00 a.m. With regard to the amount and frequency of Sundays versus workdays during which the measurements took place (more specifically: the days the participants woke up) and their possible confounding influence, we performed a chi-square analysis. A total of 860 measurements were conducted with all participants (596 with men and 264 with women). From this number, 122.86 were statistically expected to have taken place on a Sunday with all participants (85.14 with men and 37.71 with women). In reality, measurements took place on 112 Sundays with all participants (on 90 with men and on 22 with women). Thus, as Table 2 indicates, the number of Sundays for women was lower than to be expected (58.3%) and among men, the number of Sundays was slightly higher than to be expected (105.7%). However, chi-square analysis revealed that there was no significant difference in the number of Sundays compared to workdays for all participants as well as for men and women separately over the course of the overwinterings. In addition, a Pearson correlation analysis was performed to reveal any change in the relative amount of Sundays per measurement point. This analysis yielded non-significant results (p>0.05 for all participants as well as for men and women separately), indicating that there was no significant increase or decrease in the relative number of Sundays during which the measurements took place over the course of the overwinterings.

**Table 2:** Chi-square test results regarding frequency of Sundays during measurements.

Gender	Sundays (observed, expected)	Workdays (observed, expected)	Total number of measurements (observed, expected)	Chi-square value	p-value
Both	112	748	860		
	122.86	737.14	860	1.119	0.981
Male	90	506	596		
	85.14	510.86	596	0.323	0.999
Female	22	242	264		
	37.71	226.29	264	7.639	0.266

Chi-square test results regarding the frequency of Sundays versus workdays over the course of all measurements for entire study sample (n = 54, male n = 37, female n = 17).

### Statistics

Descriptive data are reported as means and standard deviations as well as median and 25^th^ and 75^th^ percentiles respectively. Anthropometric data were tested for normality and for significant differences between men and women.

Measurement data of sleep parameters and physical activity level were averaged over overwintering time for all as well as for male and female participants separately. T-test and Mann-Whitney rank sum test respectively were performed with the averaged values to investigate differences between men and women.

Analysis of covariance (ANCOVA) for all measurement data was performed with the sleep parameters being the dependent variable and the parameters gender, inhabited Neumayer Station (II or III), and overwintering season (2008 to 2014) being the covariates respectively. Age, height, body mass, body mass index, percentage of fat mass from body weight as well as physical activity level were not included as covariates because of the significant differences regarding these parameters between the sexes to avoid multicovariance.

Measurement data of the investigated parameters were plotted as scatterplots to represent one period of thirteen months, considering a maximum tolerance range of two-week time, with respect to exactly when each measurement was taken. Polynomial regression analysis was performed to track significant main effects for the dependence from overwintering time for all, as well as for male and female participants separately.

Significant linear (1^st^ order), but not quadratic (2^nd^ order) relationships, were considered to be more influenced by overall overwintering time, while significant quadratic, but not linear relationships, were considered to be more influenced by circannual changes of local sunshine radiation.

For those regression equations that yielded a significant quadratic relationship in the aforementioned regression analysis, the time difference between the maximum or minimum of these equations and the nadir (midwinter) of the local sunshine radiation (at noon averaged per two-week time increments measured in W/m^2^) was calculated. After compensation for this time difference, a linear regression analysis of these parameters and the values of local sunshine radiation was performed. Time-adjusted measurement data of these parameters were plotted as scatterplots as function of local sunshine radiation for all participants. In addition, a rank sum test was performed between the time-adjusted measurement data of these parameters for the categories of local sunshine radiation of 300 to 400 W/m^2^ versus 0 to 100 W/m^2^.

Finally, for those regression equations that only yielded a statistically significant linear, but no quadratic relationship in the regression analysis regarding the dependence from overall overwintering time, the same aforementioned linear regression analysis against the values of local sunshine radiation was performed.

All data were handled through MICROSOFT EXCEL Version 2007 (12.0.4518) and analyzed using SYSTAT SIGMAPLOT Version 13 (13.0.0.83). A two-sided p-value of below 0.05 was considered to be an indicator for statistical significance.

## Results

The overwinterers’ anthropometric data at the beginning of their respective overwintering campaign are shown in Table 3. For all parameters: age, body mass, height, body mass index, percentage of fat mass from body weight, we found a significant difference between men and women.

**Table 3:** Anthropometric parameters.

Parameter	Gender	Median	25^th^ %	75^th^ %	Rank sum test p-value
Age (years)	Both	33.0	29.8	40.3	
	Male	35.0	32.0	45.5	**<0.001**
	Female	30.0	27.0	31.0	
Body mass (kg)	Both	78.6	67.2	87.6	
	Male	83.0	76.6	92.8	**<0.001**
	Female	61.4	59.5	68.3	
Height (cm)	Both	178.0	169.5	184.0	
	Male	181.0	174.0	185.0	**<0.001**
	Female	168.0	162.0	174.5	
Body mass index (kg/m^2^)	Both	24.6	22.5	26.9	
	Male	25.9	24.4	28.2	**<0.001**
	Female	22.5	20.8	24.7	
Fat mass (%)	Both	24.8	18.3	27.6	
	Male	21.6	15.3	27.2	**0.008**
	Female	25.7	24.1	31.7	

Anthropometric parameters of the study sample (n = 54, male n = 37, female n = 17) and p-value results of Mann-Whitney rank sum test between male and female participants; bold-typed results denote significant results.

The average values of the investigated parameters over the entire overwintering time are represented in Table 4, which indicate that women had overall significantly longer average time in bed (487.7 min versus 442.7 min, p = 0.004) and longer average sleep times compared to men (389.4 min versus 355.1 min, p = 0.014).

**Table 4:** Average values of measured parameters.

Parameter	All participants (n = 54)	Male (n = 37)	Female (n = 17)	t-test p-value	ANCOVA p-value
Time in bed (min)	456.9	442.7	487.7	**0.004**	**<0.001**
	(±54.8)	(±54.2)	(±45.4)		
Sleep time (min)	365.9	355.1	389.4	**0.014**	**<0.001**
	(±47.9)	(±47.7)	(±42.0)		
Sleep efficiency (%)^†^	81.1	80.6	81.6	0.867^‡^	0.458
	(77.1, 85.9)	(76.9, 86.1)	(77.4, 85.5)		
Arousals (n/night)	9.9	9.6	10.3	0.386	**0.040**
	(±2.6)	(±2.5)	(±2.9)		
Sleep latency (min)^†^	10.6	10.1	12.4	0.301^‡^	**0.040**
	(8.6, 13.7)	(8.3, 13.8)	(9.7, 13.9)		
Sleep onset (hh:mm)^†^	12:24 a.m.	12:31 a.m.	12:04 a.m.	0.324^‡^	**<0.001**
	(11:56 p.m., 01:18 a.m.)	(11:58 p.m., 01:27 a.m.)	(11:52 p.m., 12:56 a.m.)		
Sleep offset (hh:mm)^†^	07:21 a.m.	07:15 a.m.	07:44 a.m.	0.514^‡^	0.306
	(06:58 a.m., 07:57 a.m.)	(06:57 a.m., 07:57 a.m.)	(07:04 a.m., 07:58 a.m.)		
Physical activity level (MET)	1.68	1.72	1.60	0.086	**<0.001**
	(±0.24)	(±0.25)	(±0.18)		

Sleep parameter values and physical activity level averaged over entire overwintering time, values are given as means ±sd. Sleep onset and offset are reported as times of the day.

^†^ denotes median values and 25^th^ and 75^th^ percentiles.

^‡^ denotes p-value for conducted Mann-Whitney rank sum test. ANCOVA p-values are given regarding testing of all measurement data for covariate gender; bold-typed results denote significant results.

These results are corroborated by ANCOVA, which reveal that the covariate gender significantly affected the values of the dependent variables time in bed and sleep time (both p<0.001). Although not significantly different regarding comparison of average values through t-test, ANCOVA yielded a significant difference between the sexes for the parameters number of arousals (p = 0.04), sleep latency (p = 0.04), sleep onset (p<0.001), and physical activity level (p<0.001), indicating gender to be a significant covariate influencing these parameters. The covariate inhabited station (Neumayer II below ground versus Neumayer III above ground) did not significantly affect the dependent variables (time in bed p = 0.071, sleep time p = 0.054, sleep efficiency p = 0.975, arousals p = 0.651, sleep latency p = 0.171, physical activity level p = 0.064). Only the parameters sleep onset and offset were significantly influenced by the covariate inhabited station (both p<0.001). Median sleep onset of all participants on Neumayer II was 11:24 p.m. (10:42 p.m. 25^th^ percentile, 00:32 a.m. 75^th^ percentile) versus 00:40 p.m. (11:47 p.m. 25^th^ percentile, 01:36 a.m. 75^th^ percentile) on Neumayer III. Median sleep offset of all participants on Neumayer II was 06:54 a.m. (05:50 a.m. 25^th^ percentile, 07:33 a.m. 75^th^ percentile) versus 07:27 a.m. (06:52 a.m. 25^th^ percentile, 08:09 a.m. 75^th^ percentile) on Neumayer III. The covariate overwintering season did not affect sleep efficiency (p = 0.177), arousals (p = 0.574), sleep latency (p = 0.555), and physical activity level (p = 0.128), but time in bed (p<0.001), sleep time (p<0.001), sleep onset (p = 0.006), and sleep offset (p<0.001).

**Fig 2:**
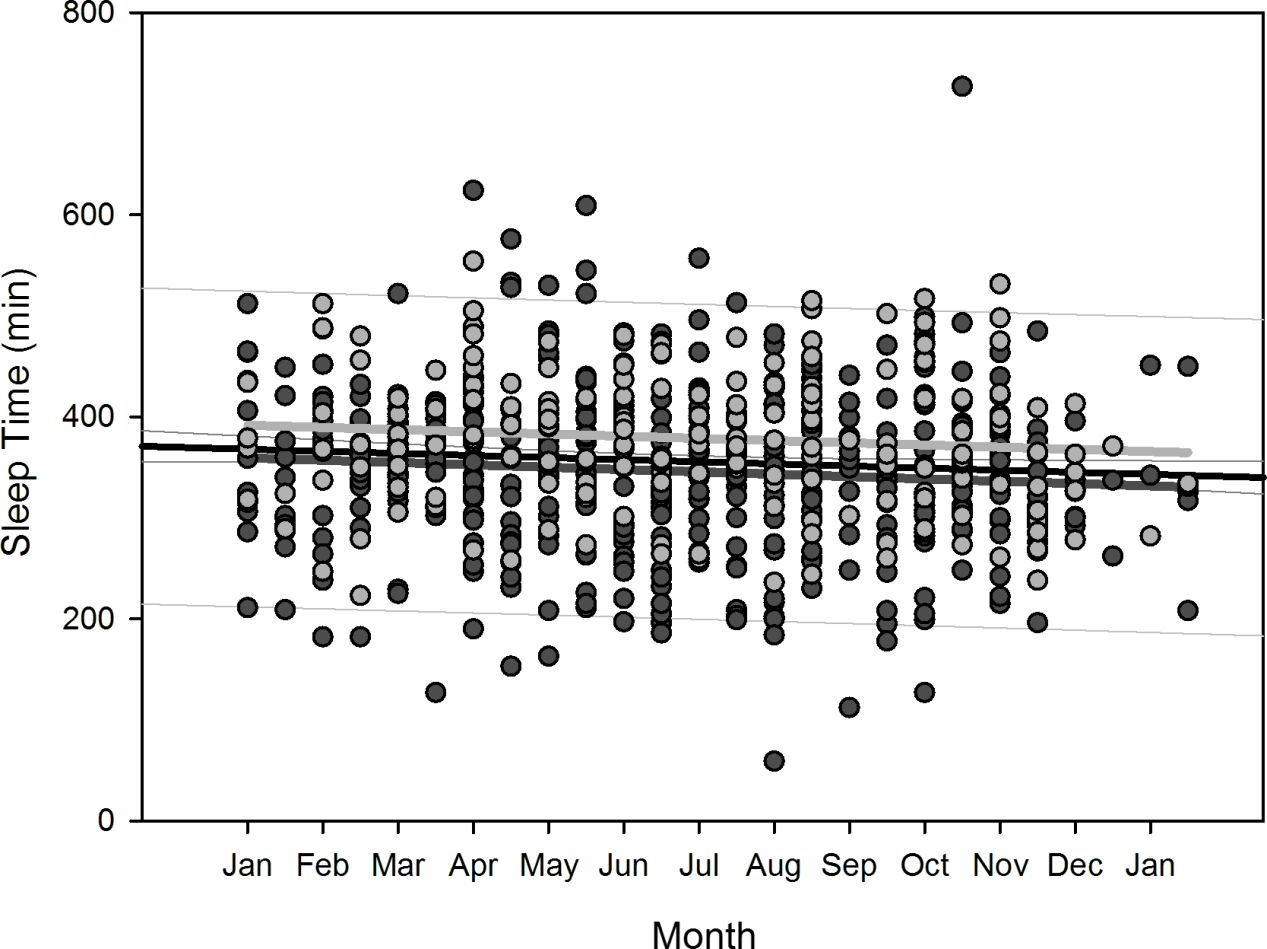
Scatterplot sleep time over overwintering time – 1^st^ order regression Changes in sleep time as function of overwintering time, scatterplot and 1^st^ order regression for all participants (black regression line with confidence and prediction intervals extended to axis) and separate for male (dark grey) and female participants (light grey).

**Fig 3:**
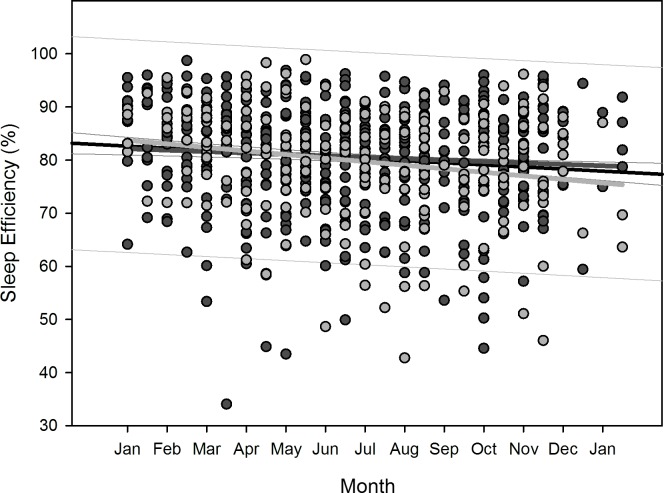
Scatterplot sleep efficiency over overwintering time – 1^st^ order regression Changes in sleep efficiency as function of overwintering time, scatterplot and 1^st^ order regression for all participants (black regression line with confidence and prediction intervals extended to axis) and separate for male (dark grey) and female participants (light grey).

**Fig 4:**
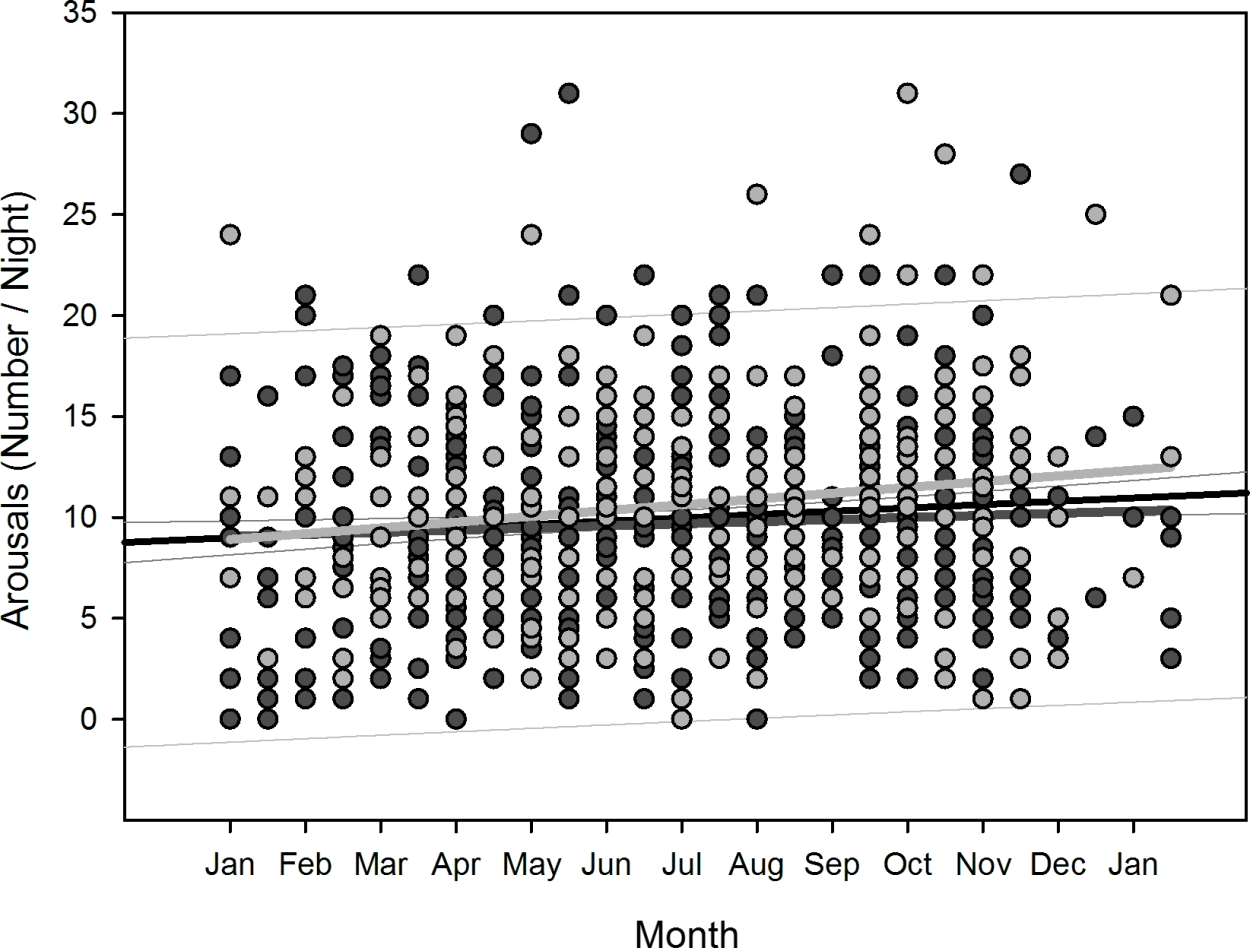
Scatterplot number of arousals over overwintering time – 1^st^ order regression Changes in number of arousals as function of overwintering time, scatterplot and 1^st^ order regression for all participants (black regression line with confidence and prediction intervals extended to axis) and separate for male (dark grey) and female participants (light grey).

**Fig 5:**
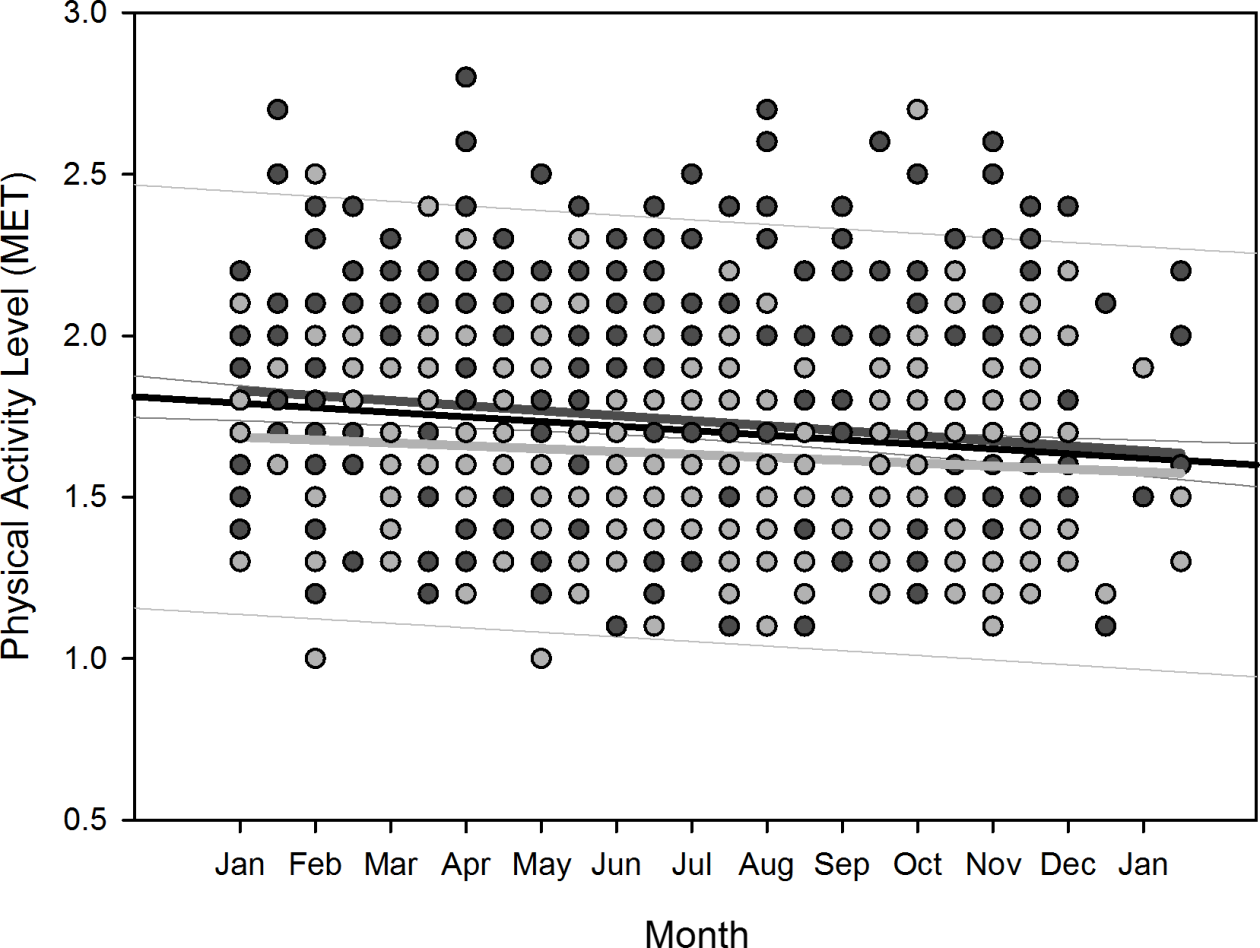
Scatterplot physical activity level over overwintering time – 1^st^ order regression Changes in physical activity level as function of overwintering time, scatterplot and 1^st^ order regression for all participants (black regression line with confidence and prediction intervals extended to axis) and separate for male (dark grey) and female participants (light grey).

Polynomial regression results are shown in Table 5. Regarding changes over overwintering time, we found two sets of dependencies: i) significant changes following a linear (1^st^ order) regression, namely sleep time for all participants (p = 0.036), sleep efficiency for all participants (p = 0.002) as well as for women separately (p = 0.006), arousals for all participants (p = 0.01) as well as for women separately (p = 0.02), and physical activity level for all participants (p<0.001) and men separately (p = 0.003); ii) significant changes following a quadratic (2^nd^ order) regression, namely time in bed for all participants (p = 0.042) as well as for women separately (p = 0.022), sleep efficiency for all (p = 0.002), male (p = 0.015), and female participants (p = 0.029), sleep onset for all participants (p = 0.01) and for men separately (p = 0.021), sleep offset for all (p<0.001), male (p<0.001), and female participants (p = 0.006), and physical activity level for all (p = 0.003) and male participants (p = 0.014). Thus, sleep time significantly decreased for all participants in a linear fashion (Fig 2) as did sleep efficiency for all and for female participants separately (Fig 3), number of arousals significantly increased linearly for all and for female participants separately (Fig 4), and physical activity level significantly decreased for all and male participants separately in a linear fashion (Fig 5).

**Table 5:** Polynomial regression results of measured parameters as function of overwintering time.

Parameter	Gender	Regression equation	Equation order	R	R^2^	p-value
Time in bed (min)	Both	= 448.860 − (0.241 * x)	1^st^	0.0159	0.0002	0.685
		= 415.991 + (6.557 * x) − (0.268 * x^2^)	2^nd^	0.1118	0.0125	**0.042**
Time in bed (min)	Male	= 441.772 − (0.799 * x)	1^st^	0.0514	0.0026	0.270
		= 417.884 + (4.218 * x) − (0.200 * x^2^)	2^nd^	0.0960	0.0092	0.144
Time in bed (min)	Female	= 470.603 + (0.629 * x)	1^st^	0.0484	0.0023	0.500
		= 410.006 + (12.724 * x) − (0.466 * x^2^)	2^nd^	0.2280	0.0520	**0.022**
Sleep time (min)	Both	= 369.208 − (1.049 * x)	1^st^	0.0816	0.0066	**0.036**
		= 361.123 + (0.625 * x) − (0.0660 * x^2^)	2^nd^	0.0877	0.0077	0.112
Sleep time (min)	Male	= 360.751 − (1.165 * x)	1^st^	0.0876	0.0077	0.060
		= 360.636 − (1.141 * x) − (0.000965 * x^2^)	2^nd^	0.0876	0.0077	0.184
Sleep time (min)	Female	= 392.848 − (1.081 * x)	1^st^	0.0989	0.0098	0.167
		= 363.209 + (4.834 * x) − (0.228 * x^2^)	2^nd^	0.1628	0.0265	0.106
Sleep efficiency (%)	Both	= 82.825 − (0.196 * x)	1^st^	0.1183	0.0140	**0.002**
		= 86.696 − (0.997 * x) + (0.0316 * x^2^)	2^nd^	0.1685	0.0284	**0.002**
Sleep efficiency (%)	Male	= 82.286 − (0.134 * x)	1^st^	0.0819	0.0067	0.079
		= 86.523 − (1.025 * x) + (0.0355 * x^2^)	2^nd^	0.1591	0.0253	**0.015**
Sleep efficiency (%)	Female	= 84.055 − (0.333 * x)	1^st^	0.1967	0.0387	**0.006**
		= 87.412 − (1.004 * x) + (0.0258 * x^2^)	2^nd^	0.2184	0.0477	**0.029**
Arousals (n/night)	Both	= 8.900 + (0.0825 * x)	1^st^	0.0987	0.0097	**0.010**
		= 8.811 + (0.101 * x) − (0.000725 * x^2^)	2^nd^	0.0988	0.0098	0.069
Arousals (n/night)	Male	= 8.996 + (0.0523 * x)	1^st^	0.0642	0.0041	0.165
		= 8.371 + (0.184 * x) − (0.00524 * x^2^)	2^nd^	0.0758	0.0057	0.246
Arousals (n/night)	Female	= 8.763 + (0.143 * x)	1^st^	0.1634	0.0267	**0.020**
		= 9.878 − (0.0800 * x) + (0.00861 * x^2^)	2^nd^	0.1741	0.0303	0.078
Sleep latency (min)	Both	= 11.420 + (0.0182 * x)	1^st^	0.0094	0.0001	0.804
		= 8.779 + (0.564 * x) − (0.0216 * x^2^)	2^nd^	0.0714	0.0051	0.067
Sleep latency (min)	Male	= 12.395 − (0.108 * x)	1^st^	0.0656	0.0043	0.155
		= 9.809 + (0.435 * x) − (0.0216 * x^2^)	2^nd^	0.0824	0.0068	0.106
Sleep latency (min)	Female	= 9.278 + (0.290 * x)	1^st^	0.1220	0.0149	0.830
		= 5.674 + (1.009 * x) − (0.0278 * x^2^)	2^nd^	0.1414	0.0200	0.154
Sleep onset (min)	Both	= 1465.842 + (0.624 * x)	1^st^	0.0400	0.0016	0.294
		= 1425.390 + (8.994 * x) − (0.331 * x^2^)	2^nd^	0.1404	0.0197	**0.010**
Sleep onset (min)	Male	= 1471.424 + (0.958 * x)	1^st^	0.0583	0.0034	0.206
		= 1428.491 + (9.975 * x) − (0.359 * x^2^)	2^nd^	0.1500	0.0225	**0.021**
Sleep onset (min)	Female	= 1449.069 + (0.171 * x)	1^st^	0.0141	0.0002	0.842
		= 1416.457 + (6.686 * x) − (0.252 * x^2^)	2^nd^	0.1292	0.0167	0.195
Sleep offset (min)	Both	= 436.322 + (0.260 * x)	1^st^	0.0200	0.0004	0.609
		= 378.321 + (12.257 * x) − (0.474 * x^2^)	2^nd^	0.2256	0.0509	**<0.001**
Sleep offset (min)	Male	= 434.479 + (0.246 * x)	1^st^	0.0173	0.0003	0.706
		= 377.585 + (12.194 * x) − (0.476 * x^2^)	2^nd^	0.2138	0.0457	**<0.001**
Sleep offset (min)	Female	= 441.502 + (0.222 * x)	1^st^	0.0200	0.0004	0.773
		= 380.090 + (12.481 * x) − (0.473 * x^2^)	2^nd^	0.2692	0.0725	**0.006**
Physical activity level (MET)	Both	= 1.799 − (0.00711 * x)	1^st^	0.1308	0.0171	**<0.001**
		= 1.896 − (0.0272 * x) + (0.000792 * x^2^)	2^nd^	0.1594	0.0254	**0.003**
Physical activity level (MET)	Male	= 1.838 − (0.00776 * x)	1^st^	0.1375	0.0189	**0.003**
		= 1.925 − (0.0260 * x) + (0.000727 * x^2^)	2^nd^	0.1597	0.0255	**0.014**
Physical activity level (MET)	Female	= 1.691 − (0.00452 * x)	1^st^	0.0959	0.0092	0.174
		= 1.810 − (0.0283 * x) + (0.000920 * x^2^)	2^nd^	0.1536	0.0236	0.122

Comparison of polynomial regression results; x denotes the time of measurement based on two-week time increments; bold-typed results denote significant results. Analyses regarding sleep onset and offset are reported as number of minutes.

**Fig 6:**
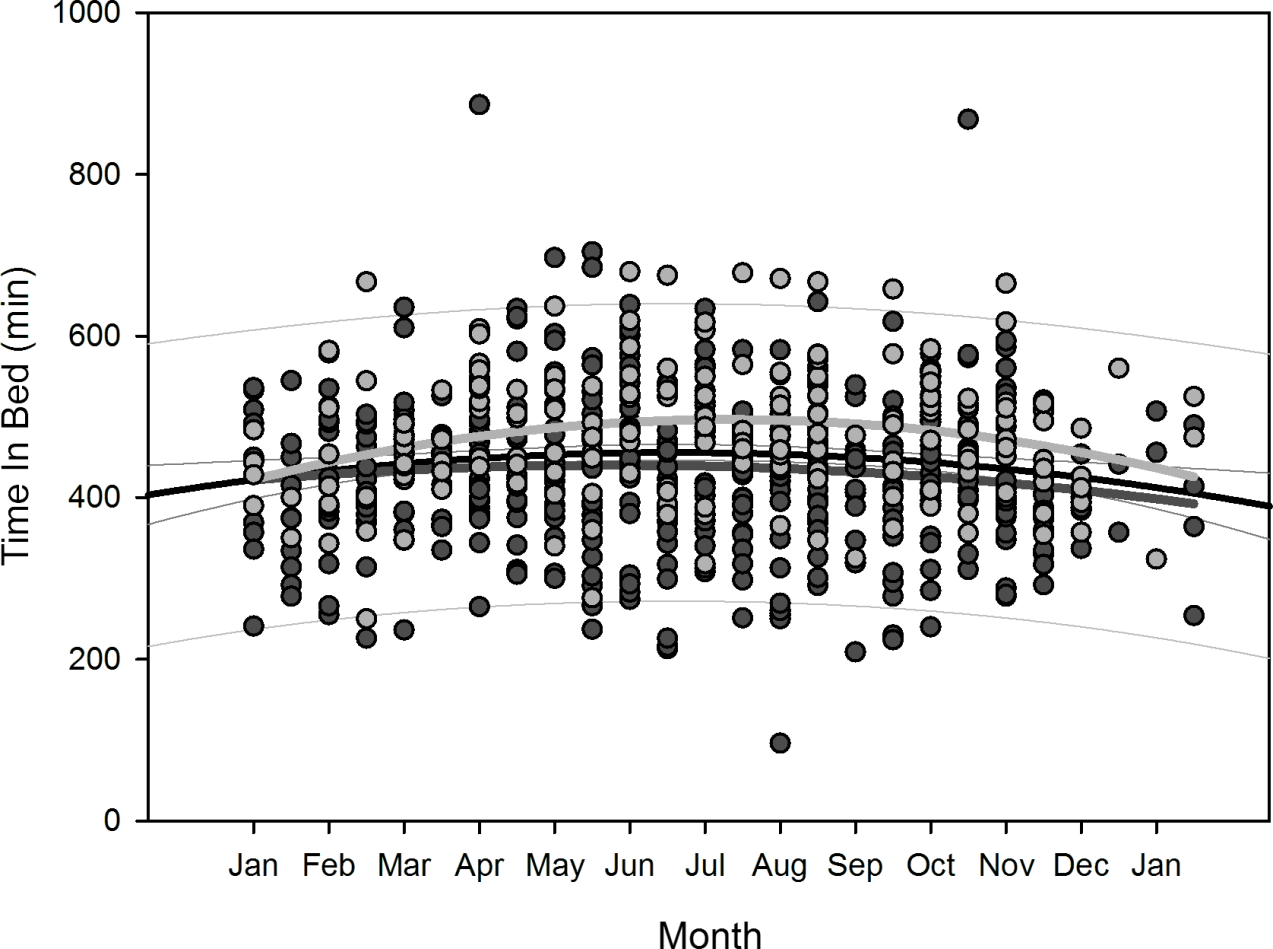
Scatterplot time in bed over overwintering time – 2^nd^ order regression Changes in time in bed as function of overwintering time, scatterplot and 2^nd^ order regression for all participants (black regression curve with confidence and prediction intervals extended to axis) and separate for male (dark grey) and female participants (light grey).

**Fig 7:**
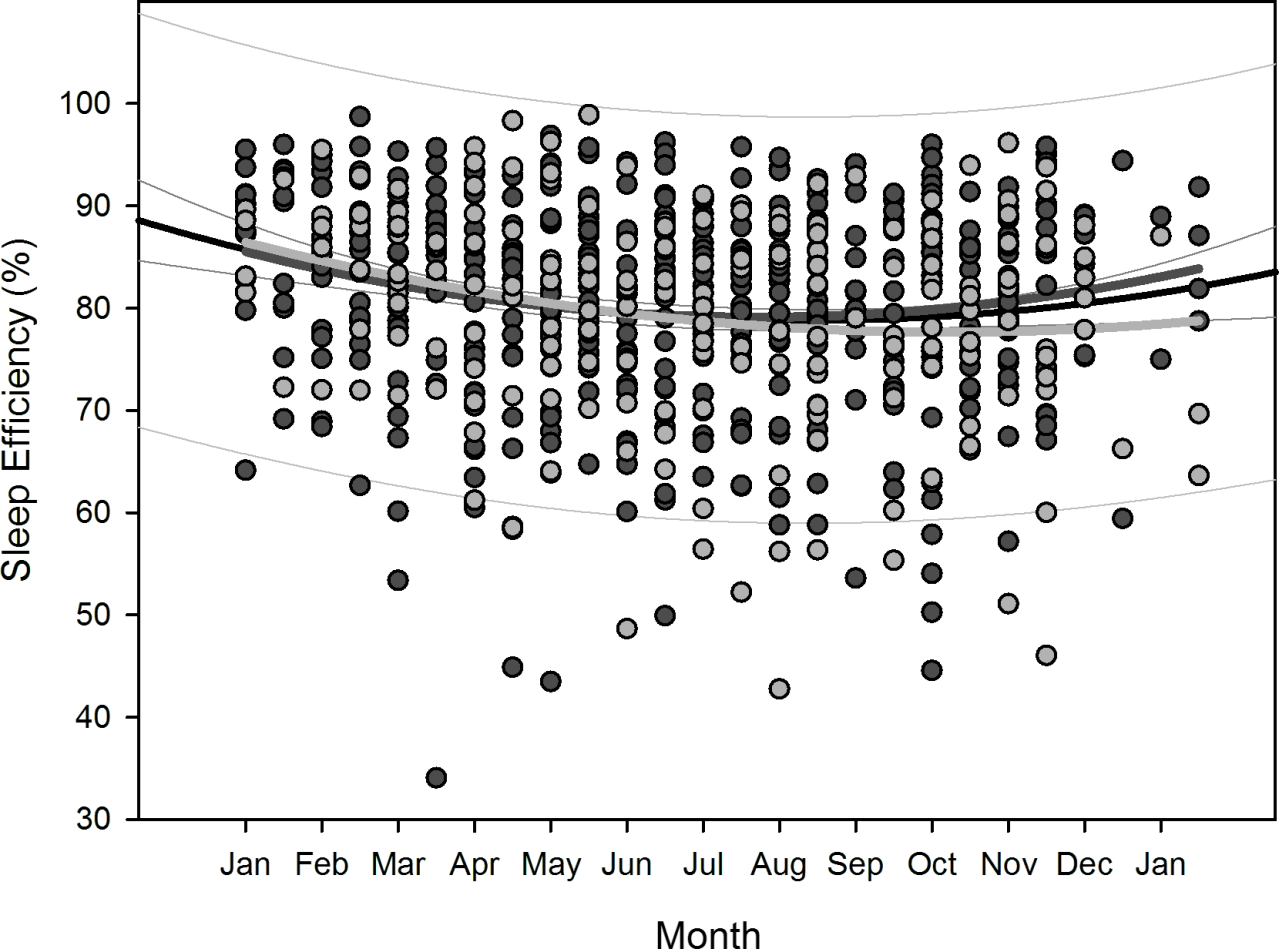
Scatterplot sleep efficiency over overwintering time – 2^nd^ order regression Changes in sleep efficiency as function of overwintering time, scatterplot and 2^nd^ order regression for all participants (black regression curve with confidence and prediction intervals extended to axis) and separate for male (dark grey) and female participants (light grey).

**Fig 8:**
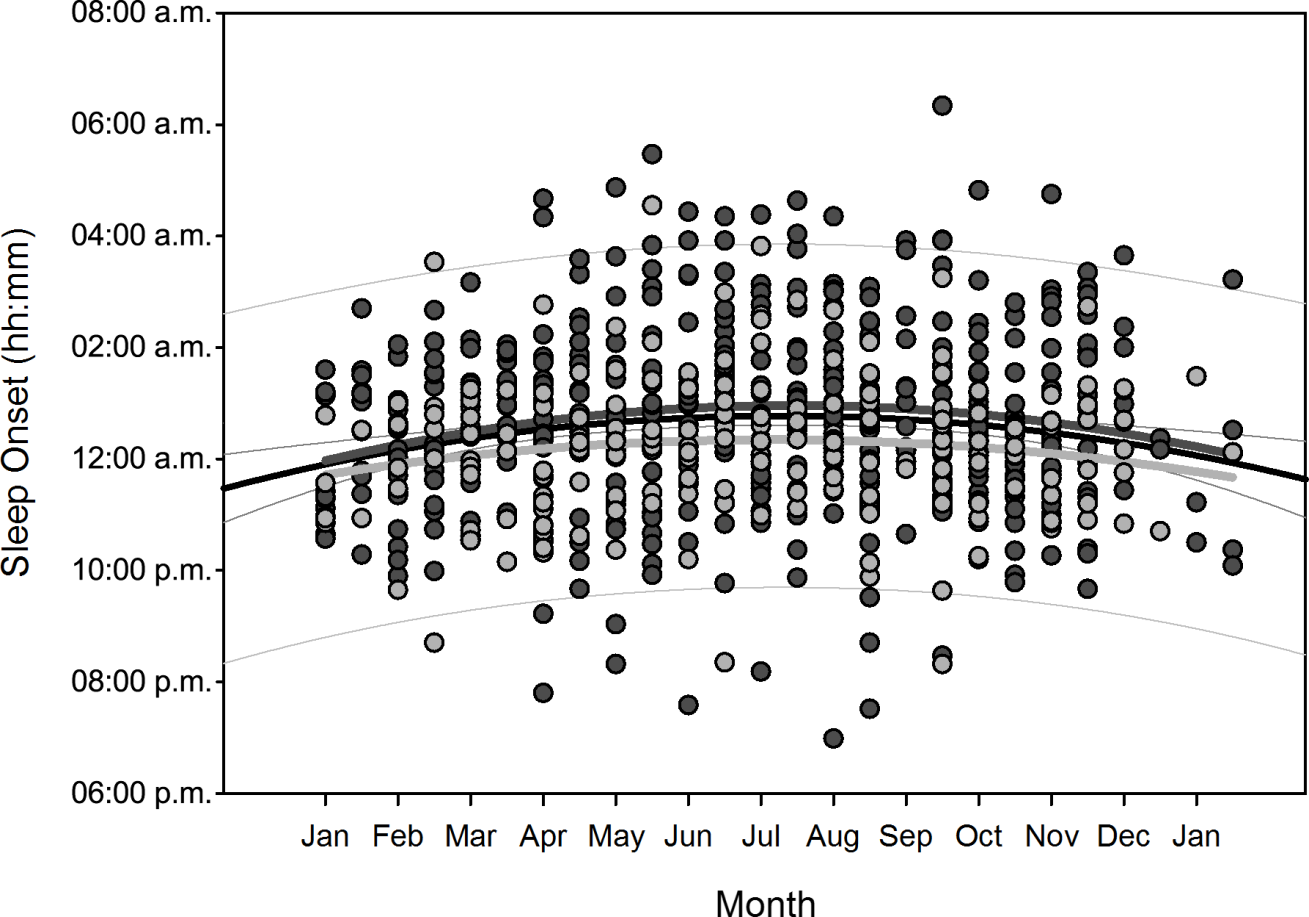
Scatterplot sleep onset over overwintering time – 2^nd^ order regression Changes in sleep onset (reported as times of the day) as function of overwintering time, scatterplot and 2^nd^ order regression for all participants (black regression curve with confidence and prediction intervals extended to axis) and separate for male (dark grey) and female participants (light grey).

**Fig 9:**
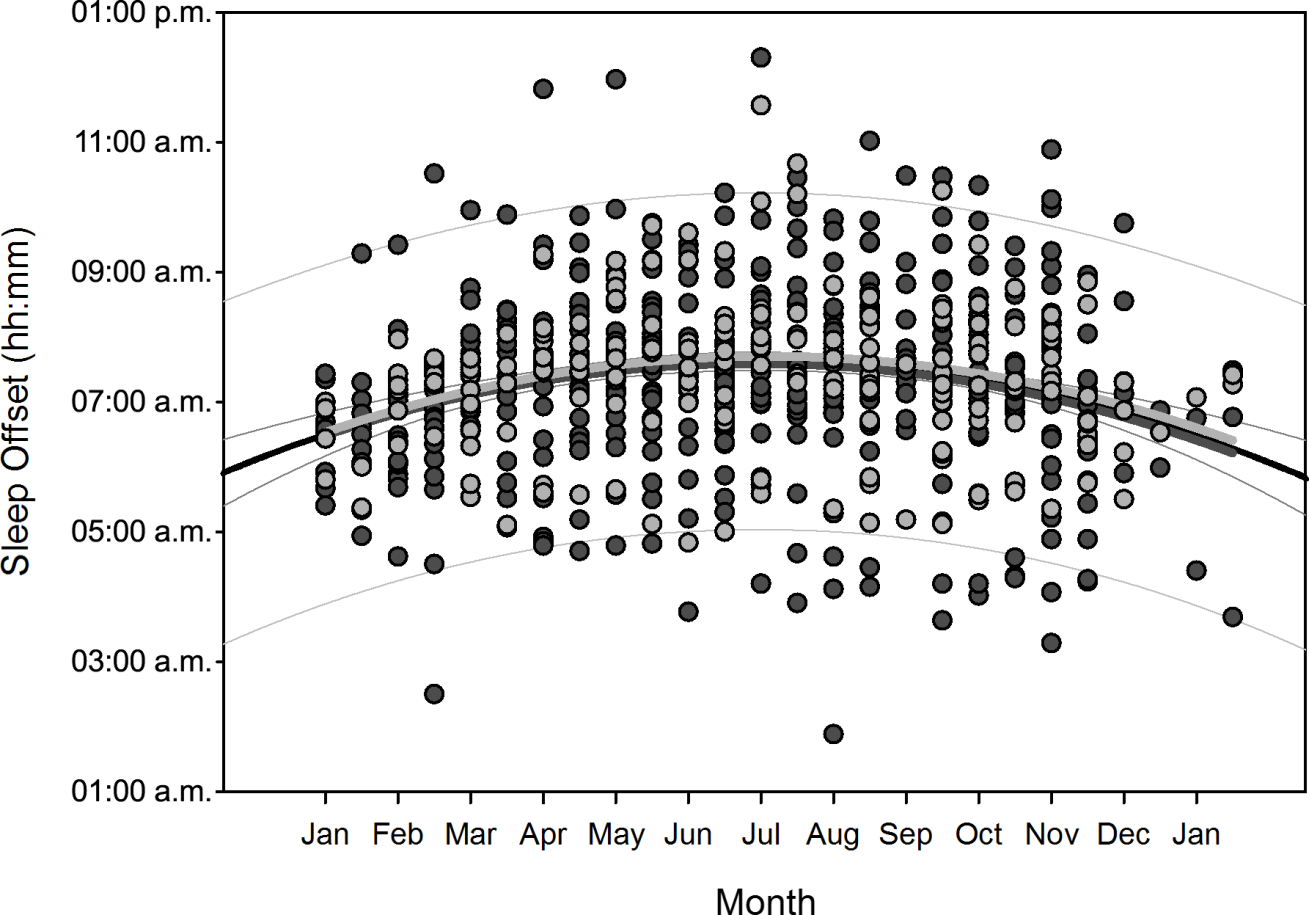
Scatterplot sleep offset over overwintering time – 2^nd^ order regression Changes in sleep offset (reported as times of the day) as function of overwintering time, scatterplot and 2^nd^ order regression for all participants (black regression curve with confidence and prediction intervals extended to axis) and separate for male (dark grey) and female participants (light grey).

**Fig 10:**
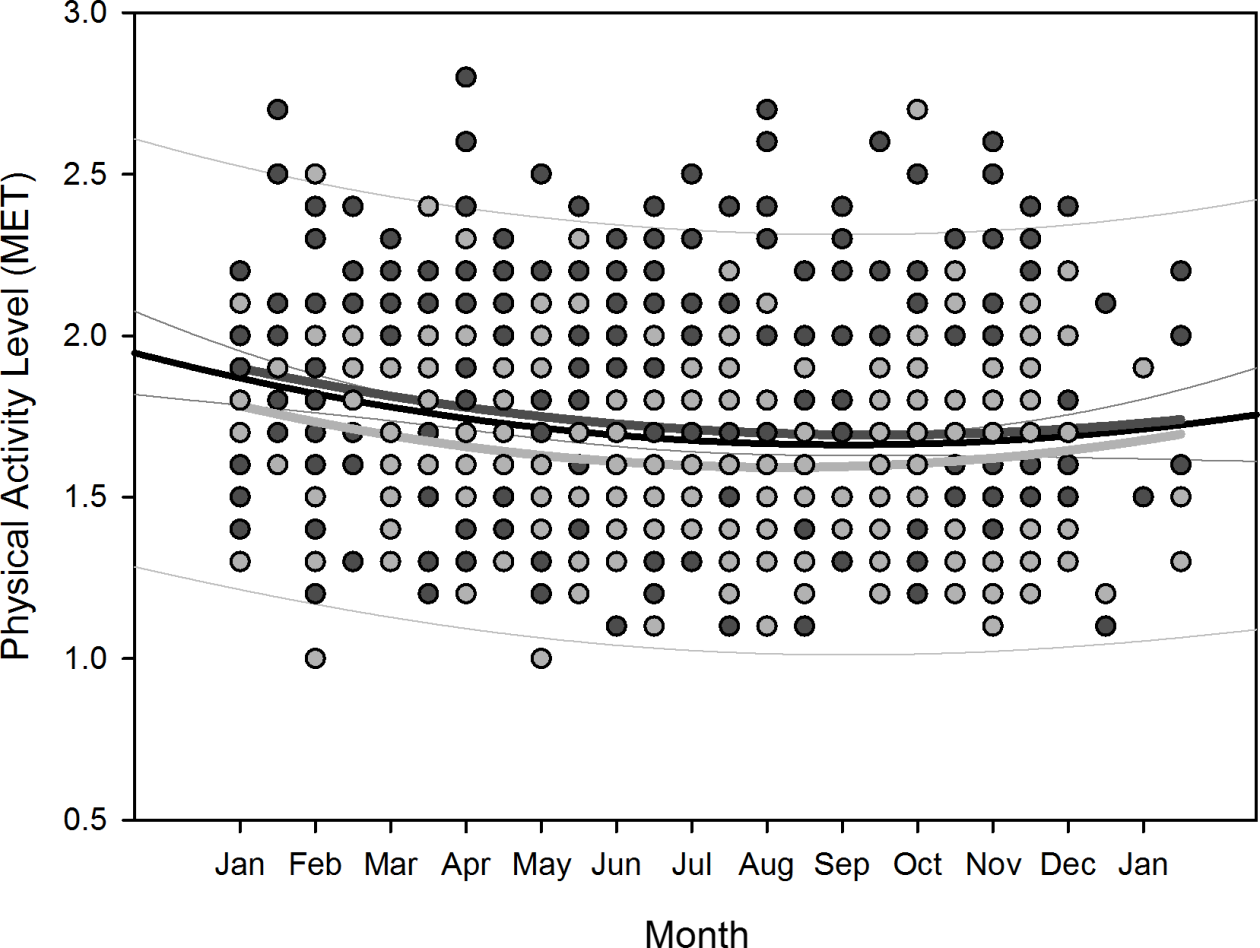
Scatterplot physical activity level over overwintering time – 2^nd^ order regression Changes in physical activity level as function of overwintering time, scatterplot and 2^nd^ order regression for all participants (black regression curve with confidence and prediction intervals extended to axis) and separate for male (dark grey) and female participants (light grey).

For the regression equations that yielded a significant quadratic relationship as function of overwintering time (i.e. time in bed for all participants as well as for women separately, sleep efficiency for all participants as well as both for men and women separately, sleep onset for all participants as well as for men separately, sleep offset for all participants as well as both for men and women separately, and physical activity level for all participants and for men separately), the calculated time delays between the respective maxima and minima to the nadir of local sunshine radiation (midwinter) are shown in Table 6. The scatterplots with the resulting negative parabola for time in bed, positive parabola for sleep efficiency, negative parabola for sleep onset and offset, and positive parabola for physical activity level are displayed in Figs 6–10. Thus, sleep efficiency not only decreased linearly over overwintering time, but also exhibited a nadir with a time delay compared to midwinter respective to Table 6. The same can be reported for parameter physical activity level with respect to all participants and men separately.

**Table 6:** Time delay of quadratic regression equations to local sunshine radiation.

Parameter	Gender	2^nd^ order regression equation	1^st^ derivative	Time delay of regression maximum or minimum to nadir of sunshine radiation (midwinter)
Time in bed (min)	Both	= 415.991 + (6.557 * x) − (0.268 * x^2^)	= 6.557 − (0.536 * x)	0.5 weeks
Time in bed (min)	Female	= 410.006 + (12.724 * x) − (0.466 * x^2^)	= 12.724 − (0.932 * x)	3.4 weeks
Sleep efficiency (%)	Both	= 86.696 − (0.997 * x) + (0.0316 * x^2^)	= -0.997 + (0.0632 * x)	7.5 weeks
Sleep efficiency (%)	Male	= 86.523 − (1.025 * x) + (0.0355 * x^2^)	= -1.025 + (0.071 * x)	5.0 weeks
Sleep efficiency (%)	Female	= 87.412 − (1.004 * x) + (0.0258 * x^2^)	= -1.004 + (0.0516 * x)	14.8 weeks
Sleep onset (min)	Both	= 1425.390 + (8.994 * x) − (0.331 * x^2^)	= 8.994 − (0.662 * x)	3.3 weeks
Sleep onset (min)	Male	= 1428.491 + (9.975 * x) − (0.359 * x^2^)	= 9.975 − (0.718 * x)	3.9 weeks
Sleep offset (min)	Both	= 378.321 + (12.257 * x) − (0.474 * x^2^)	= 12.257 − (0.948 * x)	2.0 weeks
Sleep offset (min)	Male	= 377.585 + (12.194 * x) − (0.476 * x^2^)	= 12.194 − (0.952 * x)	1.7 weeks
Sleep offset (min)	Female	= 380.090 + (12.481 * x) − (0.473 * x^2^)	= 12.481 − (0.946 * x)	2.5 weeks
Physical activity level (MET)	Both	= 1.896 − (0.0272 * x) + (0.000792 * x^2^)	= -0.0272 + (0.001584 * x)	10.4 weeks
Physical activity level (MET)	Male	= 1.925 − (0.0260 * x) + (0.000727 * x^2^)	= -0.0260 + (0.001454 * x)	11.9 weeks

Calculation of time delay of quadratic regression equations (that showed statistical significance as function of overwintering time, see Table 5) to nadir of local sunshine radiation (midwinter); x denotes the time of measurement based on two-week time increments. Analyses regarding sleep onset and offset are reported as number of minutes.

**Fig 11:**
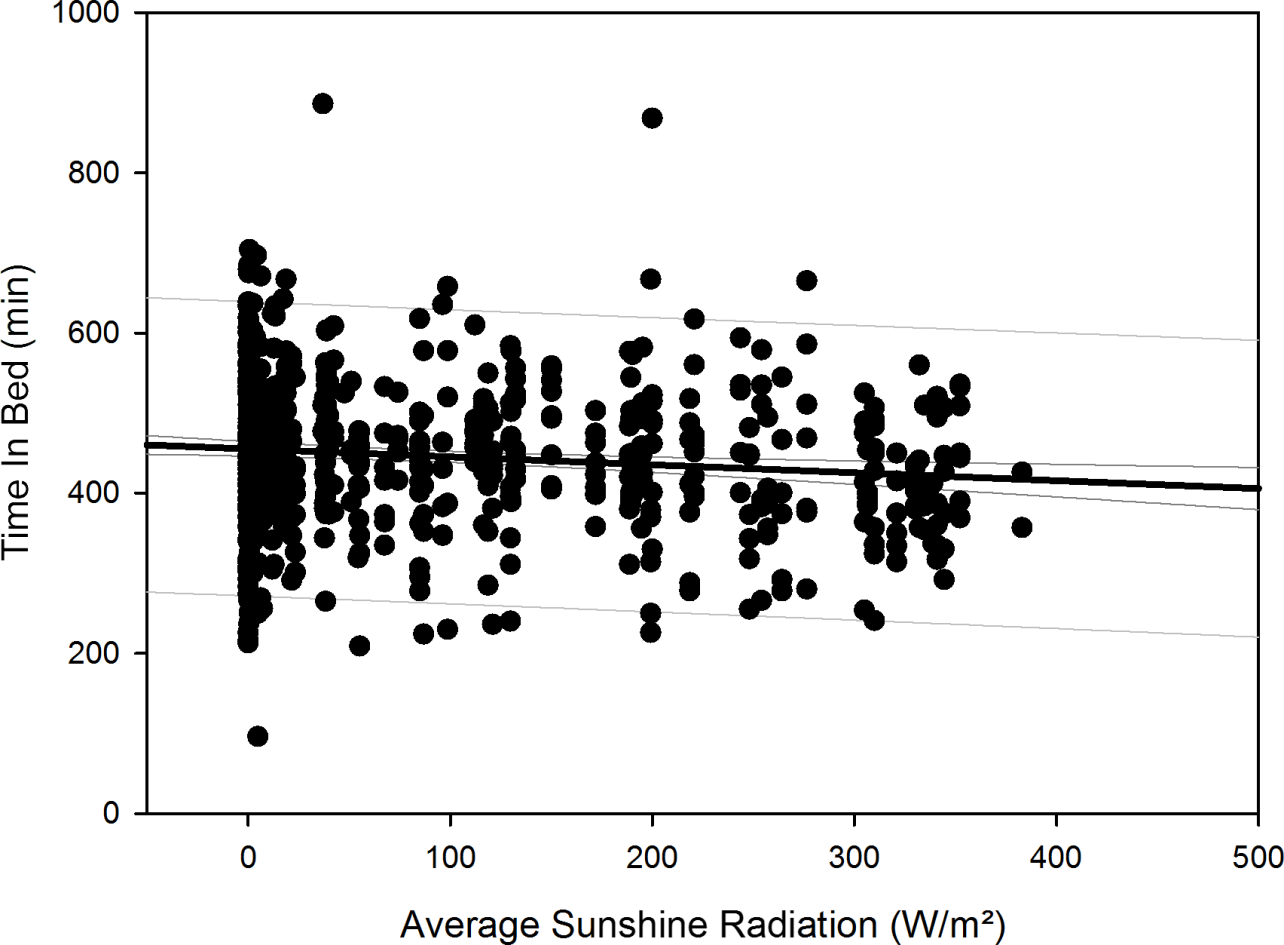
Scatterplot time in bed over sunshine radiation – 1^st^ order regression Changes in time in bed as function of average local sunshine radiation, scatterplot and 1^st^ order regression for all participants (black regression line with confidence and prediction intervals extended to axis).

**Fig 12:**
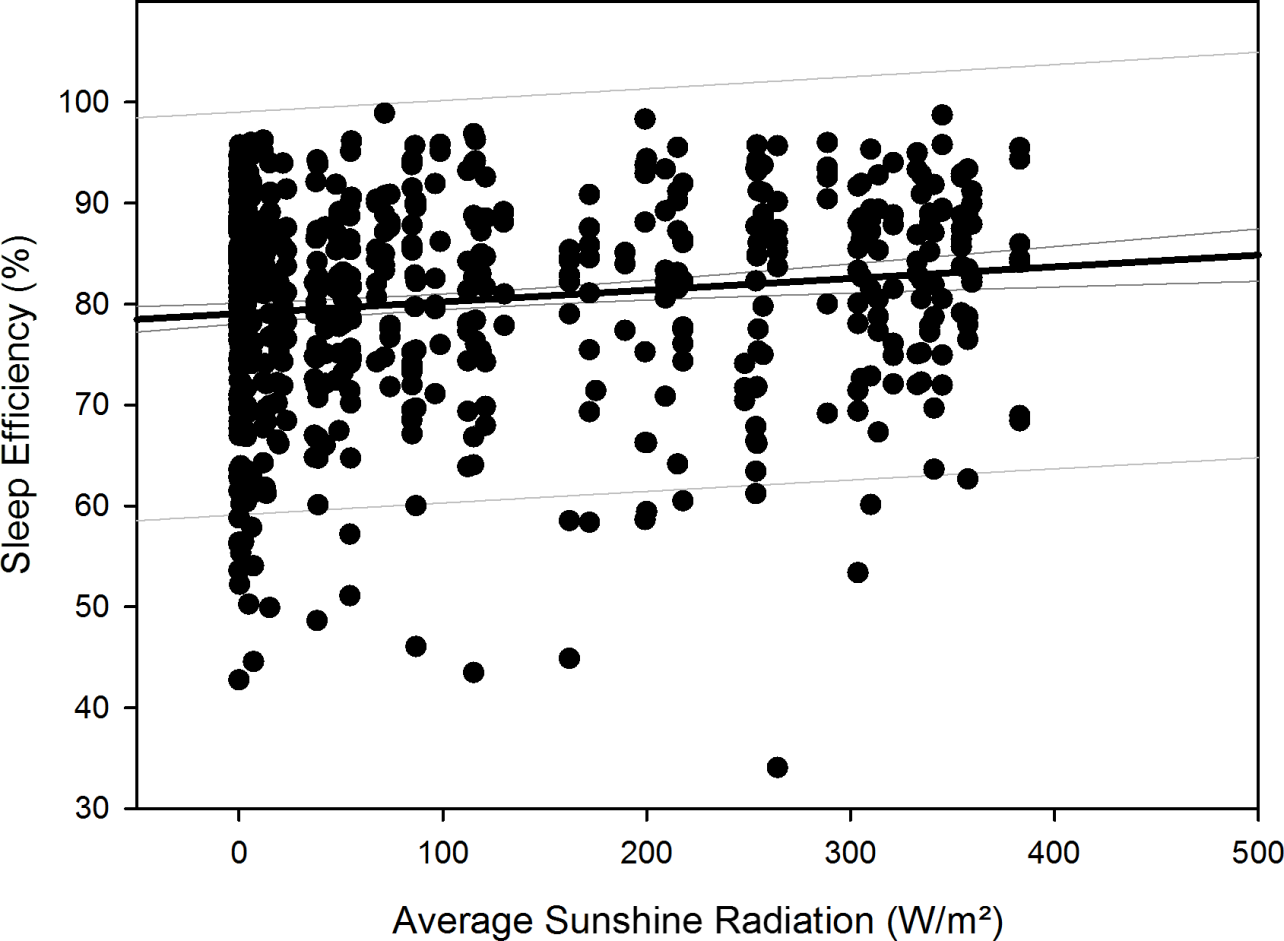
Scatterplot sleep efficiency over sunshine radiation – 1^st^ order regression Changes in sleep efficiency as function of average local sunshine radiation, scatterplot and 1^st^ order regression for all participants (black regression line with confidence and prediction intervals extended to axis).

**Fig 13:**
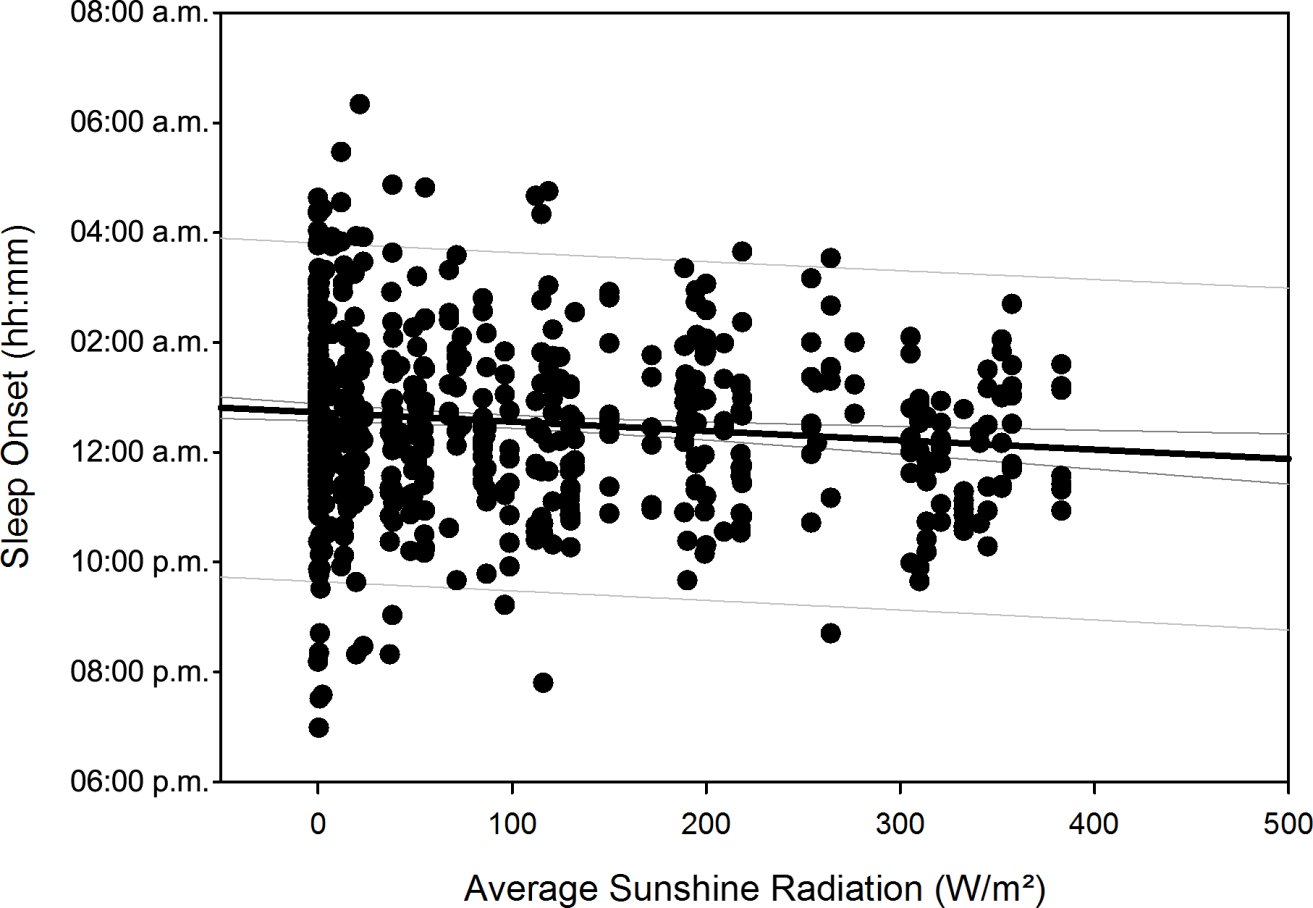
Scatterplot sleep onset over sunshine radiation – 1^st^ order regression Changes in sleep onset (reported as number of minutes) as function of average local sunshine radiation, scatterplot and 1^st^ order regression for all participants (black regression line with confidence and prediction intervals extended to axis).

**Fig 14:**
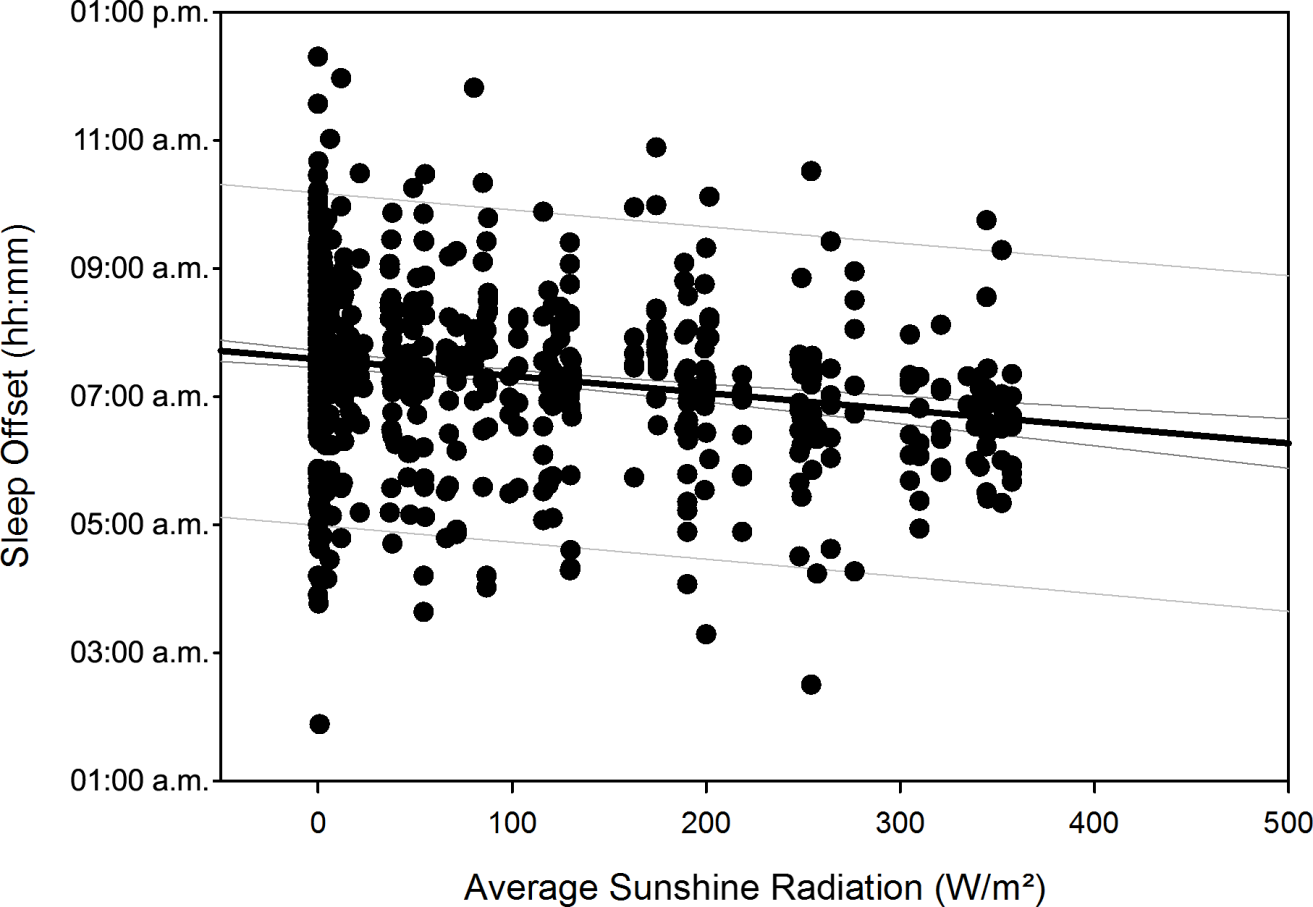
Scatterplot sleep offset over sunshine radiation – 1^st^ order regression Changes in sleep offset (reported as number of minutes) as function of average local sunshine radiation, scatterplot and 1^st^ order regression for all participants (black regression line with confidence and prediction intervals extended to axis).

**Fig 15:**
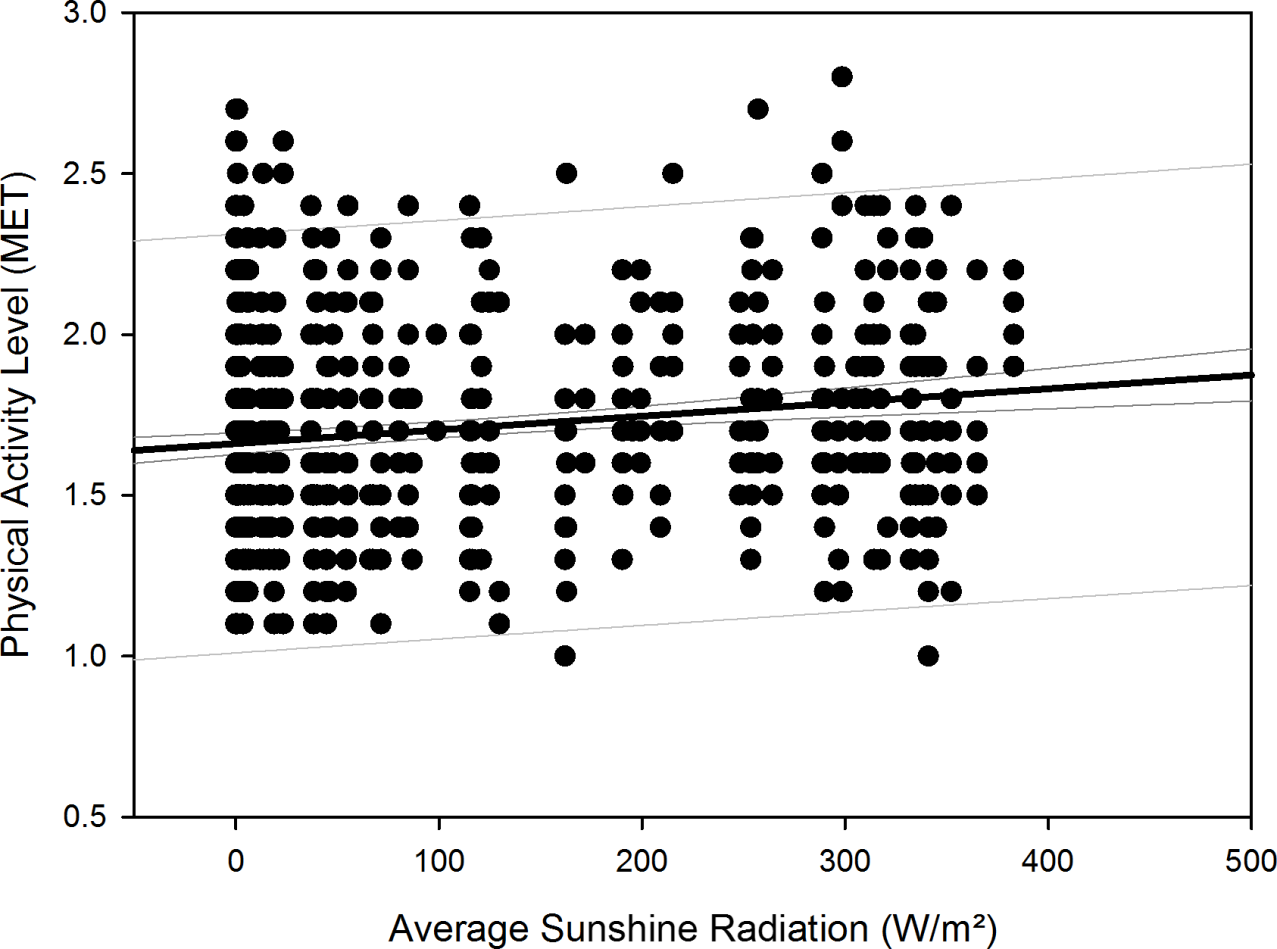
Scatterplot physical activity level over sunshine radiation – 1^st^ order regression Changes in physical activity level as function of average local sunshine radiation, scatterplot and 1^st^ order regression for all participants (black regression line with confidence and prediction intervals extended to axis).

After compensation of the respective time delays (i.e. through re-advancement of the respective measurement data that yielded the quadratic regression equations), linear regression with local sunshine radiation yielded the results shown in Table 7, indicating significant relationships with local sunshine radiation for the parameters time in bed for all participants (p = 0.002) and women separately (p<0.001), sleep efficiency for all participants (p<0.001) as well as both for men (p<0.001) and women separately (p = 0.02), sleep onset for all participants (p = 0.002) and men separately (p = 0.009), sleep offset for all participants (p<0.001) as well as both for men (p<0.001) and women separately (p<0.001), and physical activity level for all participants (p<0.001) and for men separately (p<0.001). Time-adjusted measurement data of time in bed, sleep efficiency, sleep onset, sleep offset, and physical activity level as function of local sunshine radiation are displayed as scatterplots for all participants in Figs 11–15.

**Table 7:** Linear regression of time-adjusted parameters as function of local sunshine radiation.

Parameter	Gender	Regression equation	R	R^2^	Linear regression with local sunshine radiation p-value
Time in bed (min)	Both	= 455.499 − (0.0996 * x)	0.120	0.0145	**0.002**
Time in bed (min)	Female	= 496.031 − (0.183 * x)	0.247	0.0612	**<0.001**
Sleep efficiency (%)	Both	= 79.071 + (0.0116 * x)	0.140	0.0196	**<0.001**
Sleep efficiency (%)	Male	= 79.283 + (0.0136 * x)	0.153	0.0235	**<0.001**
Sleep efficiency (%)	Female	= 78.031 + (0.0130 * x)	0.165	0.0273	**0.020**
Sleep onset (min)	Both	= 1483.678 − (0.102 * x)	0.119	0.0142	**0.002**
Sleep onset (min)	Male	= 1494.470 − (0.109 * x)	0.122	0.0149	**0.009**
Sleep offset (min)	Both	= 454.951 − (0.158 * x)	0.214	0.0457	**<0.001**
Sleep offset (min)	Male	= 452.526 − (0.150 * x)	0.194	0.0375	**<0.001**
Sleep offset (min)	Female	= 460.652 − (0.176 * x)	0.279	0.0777	**<0.001**
Physical activity level (MET)	Both	= 1.661 + (0.000427 * x)	0.162	0.0263	**<0.001**
Physical activity level (MET)	Male	= 1.687 + (0.000453 * x)	0.169	0.0287	**<0.001**

Linear regression against local sunshine radiation of the measurement data that yielded statistically significant quadratic regression equations as function of overwintering time, as shown in Table 5, after compensation for their calculated time delays to the nadir of local sunshine radiation (midwinter) as shown in Table 6; x denotes the local sunshine radiation at noon (W/m^2^) averaged for two-week time increments; bold-typed results denote significant results. Analyses regarding sleep onset and offset are reported as number of minutes.

Results regarding comparison of time-adjusted measurement data of these parameters as function of local sunshine radiation of category 300 to 400 W/m^2^ versus 0 to 100 W/m^2^ are displayed in Table 8 with the results of rank sum test. Time in bed increased by 48 minutes for all participants (p<0.001) and by 73 minutes for women separately (p = 0.01). Sleep efficiency decreased by 3.8% for all participants (p<0.001) and by 5.2% for men separately (p<0.001). The decrease in sleep efficiency of 2.2% for women was not statistically significant. Sleep onset occurred 32 minutes later for all participants (p<0.001) and 42 minutes later for men separately (p<0.001). Sleep offset occurred 54 minutes later for all participants, 48 minutes later for men separately, and 60 minutes later for women separately (all p<0.001). Physical activity level decreased by 0.2 MET for all participants (p<0.001) and by 0.3 MET for men separately (p<0.001).

**Table 8:** Comparison of time-adjusted parameters as function of local sunshine radiation of categories 300 to 400 W/m^2^ versus 0 to 100 W/m^2^.

Parameter	Gender	Global sunshine radiation, category 300–400 W/m^2^	Global sunshine radiation, category 0–100 W/m^2^	Rank sum test p-value
Time in bed (min)	Both	405.0 (364.0, 454.0)	453.0 (390.8, 512.2)	**<0.001**
Time in bed (min)	Female	414.0 (390.0, 511.0)	487.0 (432.5, 539.5)	**0.010**
Sleep efficiency (%)	Both	85.3 (77.9, 89.1)	81.5 (74.2, 86.9)	**<0.001**
Sleep efficiency (%)	Male	86.9 (78.7, 91.8)	81.7 (74.4, 87.1)	**<0.001**
Sleep efficiency (%)	Female	83.4 (76.2, 88.6)	81.2 (74.1, 86.5)	0.081
Sleep onset (hh:mm)	Both	12:04 a.m. (11:05 p.m., 12:44 a.m.)	12:36 a.m. (11:39 p.m., 01:33 a.m.)	**<0.001**
Sleep onset (hh:mm)	Male	12:07 a.m. (11:10 p.m., 01:03 a.m.)	12:49 a.m. (11:43 p.m., 01:56 a.m.)	**<0.001**
Sleep offset (hh:mm)	Both	06:40 a.m. (06:03 a.m., 07:09 a.m.)	07:34 a.m. (06:58 a.m., 08:14 a.m.)	**<0.001**
Sleep offset (hh:mm)	Male	06:40 a.m. (06:03 a.m., 07:08 a.m.)	07:28 a.m. (06:54 a.m., 08:14 a.m.)	**<0.001**
Sleep offset (hh:mm)	Female	06:42 a.m. (06:03 a.m., 07:14 a.m.)	07:42 a.m. (07:12 a.m., 08:13 a.m.)	**<0.001**
Physical activity level (MET)	Both	1.8 (1.6, 2.0)	1.6 (1.4, 1.9)	**<0.001**
Physical activity level (MET)	Male	1.9 (1.6, 2.1)	1.6 (1.4, 1.9)	**<0.001**

Time-adjusted values of parameters that yielded a quadratic relationship with overwintering time (Table 5) and a linear relationship with local sunshine radiation (Table 7), displayed as median values and 25^th^ and 75^th^ percentiles, per category of local sunshine radiation and p-value results of Mann-Whitney rank sum test between both categories; bold-typed results denote significant results. Sleep onset and offset are reported as times of the day.

For the measurement data of sleep parameters that only yielded significant 1^st^ order regression equations, but no 2^nd^ order relationship, as function of overall overwintering time (i.e. sleep time for all participants and arousals for all participants as well as for women separately, Table 5), the linear regression analysis of these measurement data as function of local sunshine radiation yielded no significant results (sleep time for all participants p = 0.137, number of arousals for all participants p = 0.665, and for women separately p = 0.172).

## Discussion

The presented results reveal that substantial changes in sleep parameters during overwintering at the Neumayer Stations in Antarctica have taken place. Influence through confounding unequally distributed measurement times (e.g. more measurements on Sundays than on workdays) could be ruled out through chi-square test; in fact, there were more measurements conducted on workdays than to be expected, especially among the women in our study. In addition, the relative number of measurements taken on Sundays versus workdays did not change over the course of the overwintering time.

Regarding anthropometric parameters, we found significant differences between men and women: age, body weight, height, body mass index, and percentage of fat mass from body weight as well as a significant influence of gender on the physical activity level; therefore, these parameters were excluded as covariates from the subsequent analysis of the sleep parameters to avoid multicovariance.

As the results indicate, there are significant differences between men and women. Female participants showed overall significantly longer average times in bed as well as significantly longer average sleep times than the male participants. Average values of number of arousals and duration of sleep latency were higher among the female participants compared to men; additionally, women fell asleep earlier than men. These gender-based differences are corroborated by the significant results of ANCOVA thus indicating a lower sleep quality among the women.

ANCOVA also revealed the covariate station residence to be a significant influence on sleep onset and offset as well as the covariate season to be a significant influence on time in bed, sleep time, sleep onset and offset, which shall be discussed further below.

Regression analysis yielded significant linear relationships between overwintering time and sleep time (all participants), sleep efficiency (all participants and women separately), number of arousals (all participants and women separately), and physical activity level (all participants and men separately), indicating linear changes of these parameters as the overwintering progressed.

The significant quadratic relationships between overwintering time and time in bed (all participants and women separately), sleep efficiency (men separately), sleep onset (all participants and men separately), and sleep offset (all participants, men and women separately), indicate that these parameters seemed to be more influenced by local sunshine radiation (and the lack thereof) than by the overwintering time. Sleep efficiency (all participants and women separately) and physical activity level (all participants and men separately) seemed to be influenced by both local sunshine radiation and overall overwintering time.

The quadratic associations with overwintering time found for these parameters were corroborated by the significant linear relationships found for their time-adjusted values with local sunshine radiation. The influence of overall overwintering time on sleep efficiency for women appears to be greater than local sunshine radiation, as the results of polynomial regression analysis and rank sum test for the different local sunshine radiation categories indicate.

Sleep parameters that showed a linear, but no quadratic relationship with overwintering time (sleep time for all participants and arousals for all participants as well as for women separately), also did not yield a significant linear relationship with local sunshine radiation, indicating that these parameters appear to be more influenced by overall overwintering time and less by local sunshine radiation.

These striking results indicate that i) overall overwintering time and ii) local sunshine radiation seem to have had different influences on the different investigated parameters during the overwinterings.

All participants slept less as the overwintering progressed over time with a decrease in sleep efficiency for all participants as well as separately for women. All participants, as well as women separately, exhibited an increase in number of arousals as the overwintering progressed over time. Physical activity level declined for all participants and men separately as the overwintering progressed, but with no significant changes among the women.

Time in bed increased for all participants and for women separately and sleep efficiency decreased for all and for both sexes separately, following a quadratic relationship with respective time delays to overwintering time and thus indicating a reaction to the decline in local sunshine radiation (i.e. the darkness-phase of the Antarctic winter). For all participants, time in bed was in median 48 minutes longer and sleep was 3.8% less efficient during the darkness-phase. The decrease in sleep efficiency was greater for men (5.2%), however, the values for women during the brightness phase (category 300–400 W/m^2^) were already 3.5% lower than those of the men. The values of sleep onset for all participants and men separately, and of sleep offset for all participants as well as for men and women separately, increased in reaction to the decline in local sunshine radiation with median delays of 32 minutes for sleep onset and 54 minutes for sleep offset for all participants. Values of physical activity level decreased in reaction to the decline in local sunshine radiation for all participants and men separately. In median, the physical activity level was 0.2 MET lower in reaction to the darkness-phase, which is equal to a decline of 11% in daily energy expenditure for all participants. While it could be argued that the quadratic relationships found in our study might be due to the higher incidence of measurements from March to August (Table 1), we would expect to see such relationships for all investigated sleep parameters, which was not the case.

The findings made in this study corroborate and expand many of the previous results regarding sleep changes during isolation-studies [47,48], long-term overwinterings in the Antarctic [1,3,5,14,16,19,20], and sleep in space [49]. Our findings indicate that changes in sleep pattern appear to prevail in a modern research station, which is in contrast to the results of some previous studies [21,22]. In addition, however, our results indicate that in the setting we investigated, women appear to be more susceptible to both influences–overall overwintering time and local sunshine radiation–than men as indicated by ANCOVA (time in bed, sleep time, number of arousals, sleep latency, and sleep onset) and by polynomial regression analysis (quadratic relationship for time in bed, linear decrease in sleep efficiency, and linear increase in number of arousals). Strikingly, this investigation is one of few studies [20,25] to report considerable gender-based differences during overwintering in Antarctica. In addition, a great number of studies regarding sleep in the Antarctic only had men in their study-groups [12–17,19,22,50]. The decline in sleep quality particularly among the women in our study is in contrast to previous investigations that had indicated that women generally exhibit a better sleep quality than men, when studied in a temperate environment without the factors of Antarctic isolation [51,52].

ANCOVA revealed a gender-based difference regarding the physical activity level with somewhat lower average values among women than men. This could be attributed to a higher physical activity among the men but also to the fact that the physical activity level expressed in MET is equivalent to an oxygen consumption (and thus energy expenditure) per kilogram body weight. As the women showed significantly higher percentages of fat mass from body weight than men, it is plausible that their average physical activity level was lower compared to men [53], as fat mass is relatively hypometabolic compared to fat free mass [54]. It is interesting to note that the values of physical activity level show a significant quadratic change in response to local sunshine radiation and a significant linear decline over the overwintering for all participants and for the men separately but not for women separately. Exercise and physical activity in general have been known to positively affect sleep quality [55,56] and may be used as countermeasure during long-term isolation and confinement to counteract psycho-physiological deconditioning [57]. However, as the physical activity level among the women of our study did not decline with statistical significance over the overwintering time, we infer that the marked deteriorations of the various sleep parameters, especially among the women of our study (increase of time in bed, decline in sleep efficiency, increase in number of arousals), cannot be attributed to an attenuation of their physical activity level.

Our results therefore suggest that other circumstances inherent to the long-term overwintering in an Antarctic station–such as psycho-social isolation [17,18,58], absence of environmental stimuli [4,59], disruption of circadian rhythm [2,19,50], cold exposure [16,60], reduced atmospheric pressure in circumpolar regions [61]–may have had a greater impact on the female participants of our investigation than on the men. Especially, as has been discussed previously, interpersonal conflict and tension may be a key source of psycho-social stress prevalent in polar overwinterings [17,18,58] as well as negative affect [17,59,62]. While it has been shown that women might be more susceptible to psycho-social stress [63,64], which has been reported to provoke a greater disturbance of sleep in women [65], we would suggest this influence, which appears to increase as the overwintering progresses, to be an important contributing factor to the reduction in sleep quality (linear decrease in sleep efficiency and linear increase in number of arousals over the time of the overwintering), more notably among the female participants of our study in Antarctica. This assumption is corroborated by a study that suggested gender differences with a deteriorated sleep quality in women to be become apparent under the impression of anxiety and depression [66]. This difference between men and women may be important, when considering life and work, not only in polar research stations, but also during long-term missions in space [67].

Regarding the marked changes of time in bed, sleep efficiency, sleep onset, sleep offset, and physical activity level that have exhibited circannual changes as function of local sunshine radiation in our study, it is known for several physiological parameters to follow seasonal variations during long-duration Antarctic residency, including mood and somatic symptoms [68]. The observed phase delay of sleep onset and offset may have been caused by an interference of the natural circadian melatonin response, which has been shown to result in delayed circadian rhythm in overwinterers in the Antarctic [1,20]. Our results corroborate the findings of these previous studies and thus indicate the importance of lighting conditions during Antarctic overwintering. In addition, we would suggest that a higher workload at the beginning and the end of each overwintering season (e.g. when the crews had to adjust to the new environment and then to prepare the transfer to the next crew), with respective longer workdays and the necessity to go to bed and wake up earlier–also known as social jetlag [69]–, may have contributed to these results.

Previous studies regarding physical activity in the Antarctic indicated that the type of residence greatly influences energy expenditure (e.g. short or long term residence, local stay or traverse), with the possibility of very high values in energy expenditure [70] and that resting metabolic rate may undergo seasonal variation with increased values during the Antarctic summer [71]. Data on Antarctic long-term residence is scarce [72], however, an investigation regarding long-term isolation and confinement showed that physical activity declined during isolation [73], which we also observed in response to overall overwintering time and lack of local sunshine radiation. In addition, the marked circannual changes in physical activity we observed may in part also have been caused by a higher workload at the beginning and the end of each overwintering season due to the altered operational demands suggested above.

A set of subclinical symptoms, such as impaired cognition, negative mood, and sleep disturbances, summarized as winter-over syndrome [74], have displayed circannual variation in previous investigations [11,50]. Lack of natural sunlight has been shown to lead to disturbances of sleep and mood in a general office setting that blocks natural sunlight [75]. Furthermore, there are indications that absence of natural sunlight may lead to a combination of changes in pituitary-ovarian hormones among women [76], known to influence mood and stress response in women [77]. As we found the women in our study to be more susceptible to the circannual changes as function of local sunshine radiation, we would like to suggest a possible link to seasonal affective disorder (SAD), which has been reported to be able to lead to changes in sleep parameters [78,79] and to have a higher prevalence among women [80,81]. Our suggested link to SAD is corroborated by reports that indicated SAD to be associated with longer sleep times [82], which were significantly higher among the women in our investigation. In addition to this possible link with SAD, it was furthermore shown in another previous study that sleep disruptions may be more detrimental to mood than an otherwise reduced sleep time, e.g. caused by delayed bedtime [83]. As we found significantly higher values in number of arousals among the women in our study, this might in turn have had a negative influence on their mood, thus intensifying the overall negative effect on their sleep. In addition, a previous study had found symptoms associated with SAD to be prevalent among overwinterers in the Antarctic and that women were significantly more associated with depressive symptoms [25], which is corroborated by another study that found women to have significantly lower scores for mental health during isolation in Antarctica [20].

Although no continuous measurements could be taken regarding the individual light exposition, the measurement results regarding illuminance inside the Neumayer Station III show that these values were in median 330 lux and that they changed in correlation with the local sunshine radiation. Even though the presentation of this environmental factor was based only on a short term measurement over three days in February 2011, the amount of local sunshine radiation, to which the illuminance values were correlated to, covered very low (52.5 W/m^2^), medium (109, 229, and 370 W/m^2^), and the highest values (439 W/m^2^) to be measured at this location, thus allowing a general approximation of the lighting conditions at the station. Possible reasons that the correlation analysis yielded only a low correlation coefficient might be attributed to scatter and diffusion from clouds and general overcast [84], as well as reflection and albedo from the snow surfaces surrounding the station [85].

One interesting aspect in this regard is that the covariate station residence did not significantly influence the values of the observed sleep parameters, except for sleep onset and offset, as the crew on Neumayer Station II fell asleep earlier and woke up earlier than the overwintering crews on Neumayer III. We also found the covariate overwintering season (2008 to 2014) to be an influence on time in bed, sleep time, sleep onset and offset, indicating that the values of these parameters differed between seasons. It should be noted that while the covariate season influenced these sleep parameters, it did not influence sleep efficiency, number of arousals, and sleep latency, which appear to have been ascribed a higher relevance when evaluating sleep quality, such as through a higher number of items in the Pittsburgh Sleep Quality Index [86]. In this sense, it could be argued that parameters thought to more notably reflect sleep quality were not influenced by overwintering season. We would suggest, that differences in group norms, different priorities regarding work and rest schedules, cultural and individual influences on group decisions [59], different styles of leadership [87] as well as the different percentages of women during each season [88], may have led to these differences between seasons. Likewise, while all other sleep parameters did not significantly differ between stations Neumayer II and Neumayer III, we would attribute the influence of the covariate station residence on sleep onset and offset to the fact that only one overwintering crew (season 2008) was investigated on station Neumayer II and that this difference might also likely be caused by a sociological group effect. Furthermore, as station Neumayer II was located underground, it is conceivable that different lighting conditions may have contributed to these results as no 24-hour bright daylight reached the inside of that station during the Antarctic summer.

Thus, regarding all parameters that did not significantly differ between stations, irrespective of the correlation between illuminance and local sunshine radiation at the Neumayer Station III described above, the results indicate that the observed changes in sleep parameters also occur at the Neumayer Station II, which was located underground. A possible explanation could be that the inhabitants of both stations may have spent more time outside the stations than previously thought or planned, which may have led them to be exposed to the local sunshine radiation during the summer months with higher radiation values. In an environment with otherwise low non-photic zeitgebers such as the Antarctic, values of about 200–1000 lux [89] or even higher values of 2500 lux [1] are discussed to be required to sufficiently influence the circadian rhythm [90], likely through the suppression of melatonin secretion [91], thus contributing to the relationships we found as function of local sunshine radiation. The lack of sunshine radiation during the darkness-phase appears to have not been sufficiently offset by the use of artificial lighting within both stations, leading to the marked changes we found during the darkness-phase–contrary to the influences of artificial lighting previously discussed [92]. This is also corroborated by our recent findings on the same cohort at the Neumayer Stations regarding vitamin D serum concentrations, which also showed marked changes in response to the circannual changes of local sunshine radiation, independent of station residence [27]. However, as Figs 11–15 show, there is a somewhat larger variability of the values of the parameters time in bed, sleep efficiency, sleep onset and offset, and physical activity level during times of very low local sunshine radiation, which might have been caused by the use of artificial lighting and electronic media and their influence on different chronotypes [93]. To offset the lack of local sunshine radiation during the darkness-phase of Antarctic residence and its negative influence on sleep, possible countermeasures in future overwinterings could include the use of light sources with augmented intensities or adjusted wavelengths (e.g. increased proportions of lower wavelengths) as a beneficial influence, which has previously been shown in polar environments [1,20,94].

The effects of the observed decline in sleep quality in our investigation may lead to a decrease in exercise performance and cognitive function [95], deterioration of immune function [96] through a possible disruption of synchronization between circadian cycle and regulation of the immune system [97], and may negatively affect the development of cardiovascular diseases [98]. Chronically disrupted sleep may also lead to decreased insulin sensitivity, increased blood glucose levels and may thus lead to a higher prevalence of type 2 diabetes [99]. This interaction may become even more important as we observed a reduced physical activity in reaction to the darkness-phase, which may further combine with negative metabolic effects of decreased vitamin d-serum concentrations [100] that we recently found in the same cohort of overwinterers [27].

To strengthen the results presented in this study, future investigations regarding sleep parameters at the Neumayer Station III could adhere to a more standardized schedule of sleep measurements, as well as include other outcome measures, e.g. changes in mood, self-perceived stress, and daytime sleepiness. It would also be desirable to include measurements of individual light exposure over the entire overwintering period as well as regarding the station’s artificial illumination to assess the conclusions we infer. In addition, sleep parameters could be measured using other, more precise means than actimetry such as polysomnography. However, actimetry proved to be a reliable and accepted means of measurement in our study since circumstances necessitated that the participants performed the measurements themselves. Further research is warranted to investigate the complex interactions occurring during long-term isolated overwinterings in the Antarctic.

Our findings in conjunction with numerous previous investigations indicate a multifaceted interaction between physiological, psychological, and social effects of isolation, lack of natural sunlight, deterioration of sleep quality, and altered energy expenditure during Antarctic overwintering, which may be important especially with regard to gender-based differences. Our findings might be extended to other environments of similar conditions for which Antarctic residence is considered an analogue, like long-duration space missions [10,11]. In such an environment, however, further adverse influences like micro-gravity, onboard noise-level, operational demands, and stress factors in space would add to the effects found in our study [101–103].

We conclude that in our investigation regarding sleep parameters during long-term Antarctic isolation there are two distinct sets of interaction: i) linear changes as function of increasing overwintering time (sleep time, sleep efficiency, and number of arousals) and ii) quadratic changes as function of local sunshine radiation (time in bed, sleep efficiency, sleep onset, and sleep offset). These influences may have led to conditions inherent to isolated long-term overwintering in a modern Antarctic research station–like increased psycho-social stress and altered circadian rhythm–both to which the women of our study have appeared to be more susceptible to. Our results could be considered for future overwinterings (e.g. crew selection, mission planning, countermeasures), especially with regard to women in the light of falling gender-barriers, which may help to ensure the physical and psychological well-being of overwinterers in Antarctica.

## Abbreviations

ANCOVA: Analysis of covariance
Apr: April
Aug: August
Dec: December
Feb: February
Jan: January
Jul: July
Jun: June
Mar: March
MET: Metabolic equivalent
Nov: November
Oct: October
PSG: Polysomnography
R: Correlation coefficient
R^2^: Coefficient of determination
S: South
SAD: Seasonal affective disorder
SD: Standard deviation
Sep: September
UV: Ultraviolet
W: West
W/m^2^: Watt per square meter
